# Data-Driven Design of PLA/PBAT/MCC/HA Composites for Biodegradable Mulch Films

**DOI:** 10.3390/polym18212617

**Published:** 2026-10-22

**Authors:** Nikki Rodriguez, Wei Ding, Lekhana Sandra, Camila Martinez, Riddhi Deepak, Ilya Gelfand, Osnat Gillor, Murat Guvendiren, Lisa Axe

**Affiliations:** 1 Otto H. York Chemical and Materials Engineering Department, New Jersey Institute of Technology, 138 Warren Street, Newark, NJ 07102, USA; ncr22@njit.edu (N.R.); cm659@njit.edu (C.M.); muratg@njit.edu (M.G.); 2 Department of Data Science, New Jersey Institute of Technology, 138 Warren Street, Newark, NJ 07102, USA; 3 The French Associates Institute for Agriculture and Biotechnology of Drylands, Ben-Gurion University of the Negev, Midreshet Ben-Gurion 8499000, Israel; igelfand@bgu.ac.il; 4 Zuckerberg Institute for Water Research, Jacob Blaustein Institutes for Desert Research, Ben-Gurion University of the Negev, Sde Boker Campus, Midreshet Ben-Gurion 8499000, Israel; gilloro@bgu.ac.il; 5 Department of Biomedical Engineering, New Jersey Institute of Technology, 138 Warren Street, Newark, NJ 07102, USA

**Keywords:** biodegradable mulch films, poly(lactic acid) (PLA), poly(butylene adipate-co-terephthalate) (PBAT), biodegradable polymer composites, Elastic Net regression, data-driven materials design

## Abstract

Conventional low-density poly(ethylene) (LDPE) agricultural mulch films generate persistent plastic waste, which has motivated the development of biodegradable mulch films (BDMs) that can provide adequate performance during use while offering improved end-of-life options. Here, composite films containing poly(lactic acid) (PLA), poly(butylene adipate-co-terephthalate) (PBAT), microcrystalline cellulose (MCC), and hydroxyapatite (HA) were evaluated for their material properties and suitability as agricultural mulches. Correlation analysis and Elastic Net regression were used to examine composition–property relationships. PBAT primarily governed ductility and tensile performance, whereas MCC significantly increased water absorption because of its hydrophilic character. Water absorption was the most reliably modeled response (training R^2^ = 0.893), while mechanical property models showed limited validation performance, which indicates that composition alone could not fully predict unseen formulations. Several formulations met commercial BDM mechanical requirements. Multi-objective optimization predicted a composition of 9.3/80.7/3.5/6.5 wt% PLA/PBAT/MCC/HA. The combined experimental and modeling framework clarifies composition–property relationships in biodegradable polymer composites and demonstrates how data-driven approaches can guide the formulation of biodegradable agricultural mulch films.

## 1. Introduction

Agriculture uses an estimated 2.5 million metric tons of plastic mulch film annually [1]. Plastic mulch conserves water, promotes seed germination and root health, prevents pests and weeds, and stabilizes soil temperature [2], but the conventional materials used for mulch, low-density poly(ethylene) (LDPE), significantly contribute to global microplastic (MP) pollution [3,4]. LDPE mulch contributes significantly to MP pollution in agricultural soils [5], where persistent fragments may alter soil properties and be incorporated into the food chain via crop uptake [6]. Replacing LDPE with materials that retain the agronomic functions of plastic mulch but do not leave persistent plastic residues is therefore a central materials-design challenge for agricultural plastics.

Biodegradable polymers are promising alternatives to LDPE, with biodegradable mulch (BDM) films already available commercially [7]. Poly(lactic acid) (PLA) and poly(butylene adipate-co-terephthalate) (PBAT) are two biodegradable polymers with promising mechanical strength and ductility for mulch applications [8]. BDM application had no adverse effect on crop quality compared with LDPE, at least during relatively short growing seasons (e.g., 49 days for onions or 120 days for maize) [7,9,10,11]. However, during longer growing seasons or under conditions that accelerate degradation, premature film breakdown can reduce soil coverage and compromise moisture conservation, temperature regulation, weed suppression, and nutrient uptake. For example, a PLA/PBAT film lost more than 30% of its soil coverage during maize cultivation under tropical conditions, while accelerated breakdown in a temperate field trial was associated with 63% lower fertilizer-N uptake compared to LDPE mulch [12,13]. Thus, some commercial BDMs may lack sufficient durability to maintain their agronomic functions throughout a full growing season, particularly under intense solar irradiation, high temperatures, or heavy rainfall [12,14]. After soil incorporation, degradation can also vary considerably with soil conditions: Xu et al. [15] observed surface erosion, cracking, and hole formation in a BDM containing 70 wt% PBAT, 5 wt% PLA, and 25% additives within one month of laboratory soil burial. By contrast, black BDMs may retain their physical integrity for longer because carbon black limits UV penetration into the polymer matrix. Although black films absorb more solar radiation and can increase soil temperature, particularly when they are in close contact with the soil, their opacity also improves weed suppression [16,17].

Current BDMs often lack sufficient durability to maintain their functionality through longer growing seasons while also failing to achieve the 90% biodegradation threshold within the recommended two-year period [18,19]. Although commercially available BDMs are designed to biodegrade in soil, their degradation under environmentally relevant agricultural conditions is often slow and incomplete [9,15,19,20]. For example, Sintim et al. [20] reported that BDM degradation, quantified by surface area reduction, reached 85–99% after approximately four months in compost but only 26–83% after 36 months in soil. When normalized by exposure time, the apparent rate of surface area loss was approximately 9–33 times greater in compost than in soil, although surface area loss indicates physical disintegration rather than complete mineralization [20]. The faster disintegration of BDMs in compost is partly attributable to industrial composting temperatures of approximately 58 °C [21], which are near the glass-transition temperature (*T_g_*) of PLA (58–65 °C) and therefore enhance polymer chain mobility, water diffusion, and hydrolysis [22]. By contrast, agricultural soils generally remain between 10 and 30 °C, well below the *T_g_* of PLA. Composting tests may therefore overestimate degradation rates under field conditions [22]. Slow mineralization has also been observed in controlled soil experiments. Schöpfer et al. [23] reported 15.4% mineralization of PLA/PBAT carbon from 0.5–2 mm particles after 230 days in dry soil, while the mineralization of smaller particles (10.7%) was not statistically significant and no significant mineralization was observed under soil with a higher water concentration. Dissanayake et al. [24] similarly reported only 2.3% PBAT and 1.7% PLA degradation after 33 weeks in soil at 25 °C. Similarly, Kansara et al. [9] observed only 12% mass loss from a PBAT film after 365 days under laboratory composting conditions, compared with 64% for a multilayer PBAT film containing a thermoplastic starch core. Differences between physical disintegration, mass loss, and carbon mineralization, as well as between composting and agricultural soil conditions, illustrate the complexity of BDM decomposition. This process involves multiple concurrent mechanisms, including oxidation, chain scission, hydrolysis, fragmentation, microbial biofilm formation, enzymatic depolymerization by enzymes such as laccases and esterases, and eventual mineralization [14,19,25,26]. The relative importance of these mechanisms also changes throughout the film lifecycle: abiotic factors such as solar irradiation and water absorption largely govern BDM durability during the growing season, whereas microbial and enzymatic degradation become increasingly important after the film is incorporated into soil [27].

Ideal mulch films must remain mechanically robust during their use and then degrade once incorporated into the soil after use. One strategy for overcoming slow degradation in soil is to blend hydrophobic high-molecular-weight polymer matrices (e.g., LDPE) with low-weight, hydrophilic additives. For example, thermoplastic starch can be incorporated as an inner layer in multilayer films with external PBAT-based layers, thereby increasing biodegradability and shortening degradation time in soil [9]. Following the same rationale, microcrystalline cellulose (MCC) can be introduced as a hydrophilic, bio-based additive to promote degradation of the mulch film after soil incorporation. MCC is a purified, partially depolymerized cellulose derived from plant biomass that can enhance the biodegradability of mulch films once incorporated into soil [28]. The hydroxyl groups on the MCC backbone make it highly hydrophilic, which promotes water uptake and facilitates hydrolytic and microbial degradation [28].

Hydroxyapatite (HA) is a calcium phosphate mineral additive that serves as a nutrient source for crops and has been investigated as a slow-release fertilizer [29,30,31,32]. HA can be produced using calcium-rich food processing wastes, such as eggshells, animal bones, and seashells, together with phosphorus recovered from nutrient-rich wastewater or biomass harvested during the remediation of eutrophic waters [33,34,35,36]. Slow-release phosphorus (P) fertilizers may be effective in mitigating eutrophication by reducing P dissolution and runoff losses; with slow-release mineral formulations, reduced P transport to surface waters is expected, as compared with conventional soluble fertilizers [37]. P release and plant uptake from HA are governed by its physicochemical properties and interactions with the rhizosphere [29,30,31,32]. Particle morphology, hydrodynamic size, surface charge, and hydrophilicity influence P delivery and uptake [29], whereas crystallinity, proton availability, and complexation with root-derived organic acids regulate HA dissolution [31]. Soil microorganisms can further increase the availability of poorly soluble P, although this effect is stronger for organic P forms than for HA [29,30,31]. For example, Tang et al. [29] demonstrated that lignocellulose-modified HA fertilizer enhanced P availability and interactions within the rhizosphere, leading to improved P uptake by soybean roots.

In this study, MCC and HA were incorporated as functional additives within a PLA/PBAT matrix to develop a biodegradable BDM film formulation with enhanced functionality. The system PLA/PBAT/MCC/HA combines a polyester matrix with a hydrophilic cellulose filler and a potential P source for crops. An Elastic Net regression machine learning (ML) model was trained using a design of experiments (DoE) dataset to quantify the effects of PLA, PBAT, MCC, and HA composition on mechanical performance and water absorption. This study asked three linked questions: which components dominate mechanical durability and water uptake; how reliably can composition alone predict these responses; and can the resulting relationships identify a formulation that balances mechanical performance with increased water absorption? The resulting models were subsequently used to identify formulations that satisfy the mechanical requirements of commercial BDMs while addressing water absorption, which has been demonstrated to be an indicator of abiotic and biotic degradation potential [38,39,40]. HA concentration was considered in the formulation as fertilizer based on its established use as a phosphorus source [29,30,31,32]; however, fertilizer release and biodegradation were not directly characterized in this study. Thermal, spectroscopic, diffraction, and microscopy measurements were used to interpret the resulting composition–property relationships.

## 2. Materials and Methods

### 2.1. Materials

The materials used include Ingeo^TM^ 4032D semicrystalline high-heat film-grade PLA (NatureWorks, Minneapolis, MN, USA), Ecoflex^®^ F blend C1200 PBAT (BASF, Ludwigshafen, Germany), MCC (Sigma-Aldrich Inc., St. Louis, MO, USA), and HA (synthetic reagent grade, Sigma-Aldrich Inc., St. Louis, MO, USA). Key properties of these grades of PLA and PBAT are listed in Table 1.

All compositions are reported in mass percentages. Sample weights were measured via analytical scale (Fisher Scientific^TM^ Education ALF104, Hampton, NH, USA) with ±0.0001 g accuracy. Commercial LDPE (76.2 μm thick embossed red mulch plastic film) was purchased as a control from Adnee Store [48]. The reference commercial BDM (biodegradable mulch film, Natural Weed Control for Gardens & Farms (48″ × 50′)) is manufactured by Dubois Agrinovation (Saint-Rémi, QC, Canada) under the trade name Bio360^®^, which is composed of Mater-Bi^®^ (a product line of proprietary blends of biodegradable polymers by Italian company Novamont S.p.A.) and vegetable starch [49]. Although Mater-Bi^®^ and Bio360^®^ are reported to comply with EN 13432 [50] and ASTM D6400-23 [51], studies on a range of environmental conditions have reported variable durability and mineralization outcomes, including failure to meet durability or biodegradation requirements over a typical growing season under ambient agricultural soil conditions [7,52,53,54,55]. The commercial BDM had an average thickness of 18.3 μm (*n* = 3). All materials and samples were stored under vacuum or nitrogen conditions (ambient pressure) in a glove box (Vacuum Technology Inc., Oak Ridge, TN, USA) with oxygen and water vapor concentrations less than 300 and 0.1 ppm, respectively.

### 2.2. Design of Experiments (DoE)

A full factorial extreme vertices DoE with one center point (1 run, 7^D^), eight vertices (2^4^ = 16 runs), and three replicates for a total of 17 × 3 = 51 runs (Table 2) was developed using Minitab^®^ Statistical Software (Version 22.4.0, Minitab LLC, State College, PA, USA). Pure PLA (18^C^), pure PBAT (20^C^), pure MCC (21^C^), pure HA (22^C^), and a filler-free blend of PLA/PBAT (19^C^) were also used as controls. The factors included PLA concentration (5–30%), PBAT concentration (70–95%), MCC concentration (0–10%), and HA concentration (0–10%). Control and validation runs were only plotted for reference. The data collected were either part of the DoE design space (runs 1^D^–17^D^), control samples (runs 18^C^–22^C^), or validation samples (runs 23^V^–30^V^) (Table 2). Convex hull analysis was performed to determine whether the validation formulations were located inside or outside the convex hull defined by the experimental design formulations (runs 1^D^–17^D^). Convex hull membership was determined using a linear programming feasibility [56], with results provided in the Supporting Information.

Sample names used in figures and figure captions indicate composition. The first value represents the PBAT/PLA weight ratio normalized to the polymer fraction (i.e., excluding MCC and HA), followed by the filler concentrations (wt%) of MCC and HA. For example, 7.3–2.5 MCC–2.5 HA contains 73.75 wt% PBAT, 21.25 wt% PLA, 2.5 wt% MCC, and 2.5 wt% HA. Samples without filler designations contain only PBAT and PLA and are identified solely by their PBAT/PLA ratio. PLA and PBAT denote the neat polymers.

### 2.3. Elastic Net Regression

Elastic Net regression models were implemented in Python using the ElasticNetCV class from the scikit-learn library (Version 1.7.2, Anaconda Inc., Austin, TX, USA). Second-order interaction terms (degree of 2, interaction only, no quadratic self-terms, intercept fitting) were generated using polynomial feature expansion. Predictor variables were scaled prior to model fitting using the scikit-learn Standard Scaler, and response variables were standardized using the scikit-learn Transformed Target Regressor. Hyperparameters (scikit-learn alpha and l1_ratio) were optimized via 5-fold cross-validation over a predefined grid (alpha ∈ [10^−6^, 10^−1^]; l1_ratio ∈ [0.5, 1]) for each response. Models were trained in standardized space, and coefficients were subsequently transformed back to original units for interpretation. DoE data (runs 1^D^–17^D^) were used for model training. Control and validation data were excluded from all model training.

Elastic Net regression is a regularized linear regression technique that combines the penalties of both Least Absolute Shrinkage and Selection Operator (LASSO) regression and Ridge regression. The method is particularly useful for datasets containing highly correlated predictor variables, as it simultaneously performs variable selection and coefficient shrinkage while improving model stability and reducing overfitting. The Elastic Net objective function is defined as:

(1) $$\hat{\beta }=\arg\underset{\beta }{\min}\left[\frac{1}{2n}\sum_{i=1}^{n}{\left(y_{i}-{\hat{y}}_{i}\right)}^{2}+\lambda \left(\alpha \sum_{j=1}^{p}|\beta_{j}|+\frac{1-\alpha }{2}\sum_{j=1}^{p}\beta_{j}^{2}\right)\right]$$

where the predicted response is given by:

(2) $${\hat{y}}_{i}=\beta_{0}+\sum_{j=1}^{p}{x_{\mathrm{ij}}\beta }_{j}$$

In these equations, *n* represents the number of observations, *p* represents the number of predictor variables, *y_i_* is the experimental response for sample *i*, and *ŷ_i_* is the predicted response. The term *x_ij_* corresponds to the value of predictor variable *j* for sample *i*, while *β*_0_ and *β_j_* represent the intercept and regression coefficients, respectively. The vector of regression coefficients is represented by *β*. The regularization parameter *λ* controls the overall penalty strength applied to the regression coefficients. Larger values of *λ* increase coefficient shrinkage and reduce model complexity. The mixing parameter α determines the relative contribution of the LASSO and Ridge penalties. When α = 1, the model becomes equivalent to LASSO regression, whereas α = 0 corresponds to Ridge regression. Intermediate values (0 < α < 1) produce the Elastic Net model. The LASSO (L_1_) penalty promotes sparsity by shrinking some coefficients completely to zero, thereby performing variable selection. The Ridge (L_2_) penalty shrinks coefficients toward zero while reducing the effects of multicollinearity and improving numerical stability.

An initial estimate of 8.75/86.25/2.5/2.5 wt% PLA/PBAT/MCC/HA was used, but additional starting points were generated via uniform random sampling of independent variables (PLA, MCC, HA) within their prescribed bounds. The weight percent of PBAT was then calculated by subtracting the sum of the weight percent of the other constituents, PLA/MCC/HA, from 100. This procedure ensured that all initial estimates were physically meaningful and within the experimental design space. A total of 150 feasible starting points were generated and used to initialize independent optimization runs. Each starting point was refined using Sequential Least Squares Programming (SLSQP), a gradient-based constrained optimization algorithm, to identify a local optimum. The best fit across all runs was selected based on the highest global desirability.

A multi-response desirability function framework following the method of Derringer and Suich [57] was used to simultaneously optimize properties, performance, and behavior. The standard Derringer and Suich [57] approach was modified in our work to include additional composition-based weighting terms to maximize HA (fertilizer) and PLA (bio-based polymer backbone) concentrations. Individual responses were transformed into unitless desirability values (*d_i_*) ranging from 0 (undesirable) to 1 (fully desirable), based on the goals defined in Table 3.

*d_i_* for each response was calculated based on its predicted performance with the desirability function defined as:

(3) $$d_{i}=\left\{\begin{array}{ll} 0 & {\hat{y}}_{i}\leq L\\ {\left(\frac{{\hat{y}}_{i}-L}{U-L}\right)}^{w} & L<{\hat{y}}_{i}<U\\ 1 & {\hat{y}}_{i}\geq U \end{array}\right.$$

where *ŷ_i_* is the predicted response, L is the lower acceptable limit, *U* is the target (or upper bound), and *w* is a weighting factor that controls the curvature of the desirability function (Table 3). The overall desirability (*D_overall_*) was calculated as the geometric mean of individual *d_i_*s:

(4) $$D_{\mathrm{overall}}={\left(\prod_{i=1}^{m}d_{i}\right)}^{1/m}$$

where *m* is the number of responses included in the optimization (*m* = 5 in this work).

This approach penalizes solutions with poor performance in any individual response and promotes balanced optimization across all properties. Additional composition-based weighting terms (*w_HA_* and *w_PLA_*) were included to favor greater HA and PLA concentrations within the allowable design space:

(5) $$D_{\mathrm{final}}=D_{\mathrm{overall}}{\left(\frac{\mathrm{HA}}{10}\right)}^{w_{\mathrm{HA}}}{\left(\frac{\mathrm{PLA}}{30}\right)}^{w_{\mathrm{PLA}}}$$

where HA and PLA were normalized by their maximum allowable concentrations, 10 wt% and 30 wt%, respectively. In this work, *w_HA_* = *w_PLA_* = 1, which indicates equal emphasis on increasing HA and PLA concentration in addition to maximizing the response-based desirability. Model performance was evaluated using the coefficient of determination (R^2^) and normalized root mean squared error (*nRMSE*). The R^2^ (Equation (6)) value quantifies the proportion of variance explained by the model, while *nRMSE* (Equation (7)) represents the average prediction error normalized by the response range and expressed as a percentage.

(6) $$R^{2}=1-\frac{\sum_{i=1}^{n}(y_{i}-{\hat{y}}_{i}{)}^{2}}{\sum_{i=1}^{n}(y_{i}-\bar{y}{)}^{2}}$$

The *nRMSE* enabled comparison among responses with different units and orders of magnitude (e.g., water absorption and *ε_Break_*).

(7) $$\mathrm{nRMSE}=\frac{\sqrt{\frac{1}{n}\sum_{i=1}^{n}(y_{i}-{\hat{y}}_{i}{)}^{2}}}{y_{\max}-y_{\min}}\times 100\%$$

In these equations, *n* is the total number of observations, *y_i_* is the experimentally measured value for observation *i*, *ŷ_i_* is the corresponding model-predicted value, and *ȳ* is the mean of all measured values. In Equation (7), *y_max_* and *y_min_* represent the maximum and minimum measured values in the dataset, respectively. The term *∑*(*y_i_* − *ŷ_i_*)^2^ represents the residual sum of squares, which quantifies the prediction error of the model. The term *∑*(*y_i_* − *ȳ*)^2^ in Equation (6) represents the total sum of squares, which quantifies the overall variability of the experimental data. Validation formulations were manually selected from the feasible mixture space based on the predicted location of the optimum, before modeling was performed. Only formulation 30^V^ corresponded to the composition calculated directly by desirability optimization and then prepared for characterization to evaluate predicted properties.

### 2.4. Film Preparation

Films were prepared using commercial feedstocks of polymer resin pellets (PBAT, PLA) and powder additives (MCC, HA). The particles were first suspended (“pre-mixed”) in the polymer matrix by heating them at 170 °C in the hot melt press without applying force. This step was critical in reducing losses during loading of the powders (MCC, HA) into the hot melt mixer, thus preserving concentrations added. Without this step, loading the powder into the mixer was not possible due to powder lifting while loading into the small mixing chamber. For consistency, blends without powder additives (e.g., pure PLA) were also subjected to this “pre-mixing” step before melt mixing. The “pre-mixed” blends/composites were then loaded into the hot melt mixer. A HaakeBuchler Rheocord 40 (Thermo Fisher Scientific, Waltham, MA, USA) double-arm mixing unit was used to mix batch sizes of 40 g with roller mixing blades at 180 °C and 55 rpm until the torque stabilized (<10 min for all blends). The melt-mixed blends/composites were subsequently loaded into a bronze cavity (13 cm diameter, 0.033 cm height) for hot melt pressing via hydraulic press (Carver Laboratory Press, Wabash, IN, USA) at 0.84 MPa and 170 °C for 90 s. Films were cooled to room temperature using cold water within 5 min at a rate of approximately 1.5 °C·s^−1^. The experimental protocol was adapted from our previous publication [8]. A 7326A (Mitutoyo, Kawasaki City, Kanagawa, Japan) dial thickness gauge (±0.005 mm) or a 700-118-30 (Mitutoyo, Kawasaki City, Kanagawa, Japan) digital thickness gauge (±0.001 mm) was used to measure film thickness prior to mechanical characterization.

### 2.5. Mechanical Testing

Mechanical testing was performed with an Instron^®^ 5967 (Instron^®^, Norwood, MA, USA) or Instron^®^ 68FM-300 (Instron^®^, Norwood, MA, USA) fitted with tapered grips. ASTM Standard D 638-22 [58] Type V dog bones were cut with ASTM-certified cutting dies (Fremont Cutting Dies, Inc., Fremont, OH, USA). Elongation at break (*ε_Break_*) and strength at break (*σ_Break_*) were characterized in triplicate for each sample from engineering stress–strain curves obtained at 50 mm·min^−1^ testing speed and room temperature. Replicates were collected from subsamples cut alongside the same direction from the same processed film. Accordingly, the statistical analysis was used to characterize intra-film variability and differences among formulations, while recognizing that specimens originating from the same film are not statistically independent experimental batches [59]. Calculations and testing conditions followed guidance from ASTM Standard D 638-22 [58] (see Supporting Information). All dog bone replicates were covered with tape in the gage regions to prevent slipping during stretching. Samples were characterized in the pristine state, after UV aging, and after submersion in water for 80 days (saturated in water, only for DoE and control samples). Compact letter display using Tukey’s Honestly Significant Difference (HSD) was used to display significant differences (95% confidence) between a pristine sample, a sample exposed to UV aging, and a sample that was saturated in water for 80 days. For a given composition, significance was calculated between the means of the pristine, UV-aged, and water-saturated samples (*n* = 3). The letters displayed (above the result in the figure) within a composition have no relation to those displayed for another composition and only reflect groups based on intra-composition treatment (i.e., pristine and UV-aged versus saturated with water). Mechanical properties for samples after their saturation in water were not calculated for the validation dataset, as they were not used in predictions. Data were smoothed prior to calculating *ε_Break_* and *σ_Break_* using a Savitzky–Golay filter with a window length of 9 points and polynomial order of 3. Analyses (including Savitzky–Golay smoothing) were performed using Python (Version 3.10.9, Anaconda Inc., Austin, TX, USA) SciPy library.

### 2.6. Ultraviolet (UV) Accelerated Aging

UV aging experiments were conducted in a laboratory fume hood equipped with air circulation to remove reactive gases, eight T12 fluorescent tube 340 nm light sources (Q-Lab Corporation, Westlake, OH, USA), and two IONBOARD S22 LED Grow Light Boards with Samsung LM301H Diodes (AC Infinity, City of Industry, CA, USA). Accessories were programmed on an 8 h on/4 h off cycle, in accordance with ASTM Standard G 154-23 [60]. Temperature was recorded using a Controller 69 Pro (AC Infinity, City of Industry, CA, USA) fitted with a temperature probe. A film of each sample was used, and three dog bones were cut from each sample for mechanical analysis after aging. Cumulative UV irradiation, H (J·m^−2^), is calculated using irradiance, E (W·m^−2^), and irradiation time, t (s) (Equation (8)):

*H* = *E* ⋅ *t* (8)

Irradiance was measured using a UV513AB (General Tools & Instruments, Secaucus, NJ, USA) 280–400 nm light meter with ±4% resolution. All samples were maintained 20 cm from the light source, where irradiance was measured and averaged before calculating the experiment duration. Exposure time was calculated to simulate 3 months of summer irradiance using Rutgers New Jersey Weather Network irradiance data [61]. Calculations are provided in Supporting Information. Samples that were not exposed to accelerated aging are labeled “pristine”, while subjected samples are denoted “UV-aged” or “aged” (used interchangeably). Film mass was recorded prior to exposure (m_0_) and then after exposure (*m_t_*) to calculate mass change (Δ*m*) (Equation (9)).

(9) $$∆m=\frac{m_{t}-m_{0}}{m_{0}}\times 100\%$$

### 2.7. Thermogravimetric Analysis (TGA)

Degradation parameters, moisture concentration, and char yield were evaluated using TGA on a Thermogravimetric Analyzer TGA 8000 (PerkinElmer Inc., Waltham, MA, USA). Degradation parameters include the temperature of maximum degradation (T_Max_) and the temperatures at 10% mass loss (90% remaining mass, T_90_), 15% mass loss (85% remaining mass, T_85_), and 50% mass loss (T_50_). T_85_ is considered the decomposition onset [62]. Samples were loaded into a platinum pan with approximately 10 mg sample loading per run. The temperature program was performed under 20 mL·min^−1^ nitrogen gas flow as follows: isotherm for 5 min at 30 °C followed by heating from 30 °C to 600 °C at 10 °C·min^−1^. TGA was not conducted on the validation dataset, as it was not used in predictions.

### 2.8. Water Absorption

Water absorption was measured in triplicate using guidance from ASTM Standard D 570-22 [63]. Films were cut to 80 mm squares and submerged in deionized water equilibrated to atmospheric conditions inside glass or poly(styrene) Petri dishes. O-rings were used to keep the films submerged. We selected 80 mm squares because they are of sufficient mass to detect weight changes using our ALF104 (Fisher Scientific Education, Waltham, MA, USA) analytical scale with ±0.0001 g accuracy. The pH of the water was 5.72 ± 0.06 (*n* = 5) under atmospheric conditions, measured using an Accumet AB250 (Fisherbrand, Waltham, MA, USA) pH meter. Water absorption was measured until there were three consecutive weight changes < 5 mg or <1 wt% of the total weight increase (whichever was greater), in accordance with ASTM Standard D 570-22 [63]. After equilibration, samples underwent mechanical testing as outlined in Section 2.5.

### 2.9. Fourier-Transform Infrared Spectroscopy (FTIR)

FTIR was used to probe quantitative and qualitative signatures of degradation. FTIR traces were obtained using a Cary 620 (Agilent Technologies, Inc., Santa Clara, CA, USA) or Cary 630 (Agilent Technologies, Inc., Santa Clara, CA, USA) via diamond Attenuated Total Reflectance (ATR). Spectra of each sample were collected from 32 scans with 2 cm^−1^ resolution and air background subtraction. The carbonyl index (CI) and carboxylic acid index (CAI) were used as indicators of degradation [64]. CI and CAI are calculated using Equation (10):

(10) $$\mathrm{Index}=\frac{{\mathrm{Area}}_{\mathrm{Functional}\;\mathrm{Group}}}{{\mathrm{Area}}_{\mathrm{Reference}\;\mathrm{Group}}}$$

where the functional group is either the C=O or the COO-H group and the reference peak is the methylene CH_2_ group in the polymer backbones. Wavenumber regions were modified slightly from that used by Tanjung et al. [65] and Graf et al. [19] for similar materials to include the exact peaks found in our samples (Table 4).

### 2.10. X-Ray Diffraction (XRD)

XRD was characterized using a PANalytical Empyrean (Malvern Panalytical Ltd., Almelo, The Netherlands) X-ray diffractometer with a 27 mm monocrystalline Si zero-background sample holder operated at 45 kV and 40 mA or a MiniFlex 600 X-ray diffractometer (Rigaku Corporation, Akishima, Tokyo, Japan) operated at 40 kV and 15 mA. Both instruments used Cu Kα radiation (λ = 1.5438 Å) sources. Percent crystallinity (*χ*) was calculated using the following equation:

(11) $$\chi =\frac{I_{\mathrm{crystalline}}}{I_{\mathrm{amorphous}}+I_{\mathrm{crystalline}}}\times 100\%$$

where I_crystalline_ and I_amorphous_ are the areas under the crystalline and amorphous signals of the XRD diffractograms, respectively [66]. Note that the instrument background signal (“constant background signal”) was subtracted from all diffractograms prior to calculation of *χ*. The crystallite size (*D*) was calculated using the Scherrer equation below:

(12) $$D=\frac{K\cdot \lambda }{\beta \cdot \mathrm{cos\theta}}$$

where *β* is the peak’s full width at half max (FWHM) in radians, λ = 0.15438 nm, *θ* is the Bragg angle (peak position) in radians, and *K* is the shape factor, assumed to be 0.9 for PLA, PBAT, and HA crystallites [67,68] and 0.94 for MCC crystallites [69]. XRD was not characterized for the validation dataset, as it was not used in predictions.

### 2.11. Scanning Electron Microscopy (SEM)

Film topography and particle size of MCC and HA were characterized using JEOL JSM-7900F (JEOL USA Inc., Peabody, MA, USA) field emission SEM and 10 nm 80/20 wt% Au/Pd to minimize charging. Particle sizes were analyzed using Fiji ImageJ (Version 1.54p, National Institutes of Health, Bethesda, MD, USA) software. Because the analyzed particles exhibited mostly non-uniform morphologies, particle size was reported as the Feret diameter rather than an equivalent circular diameter. This approach captures the maximum projected dimension of irregular particles and enables consistent comparison across samples.

## 3. Results and Discussion

### 3.1. Mechanical Testing

**Figure 1:**
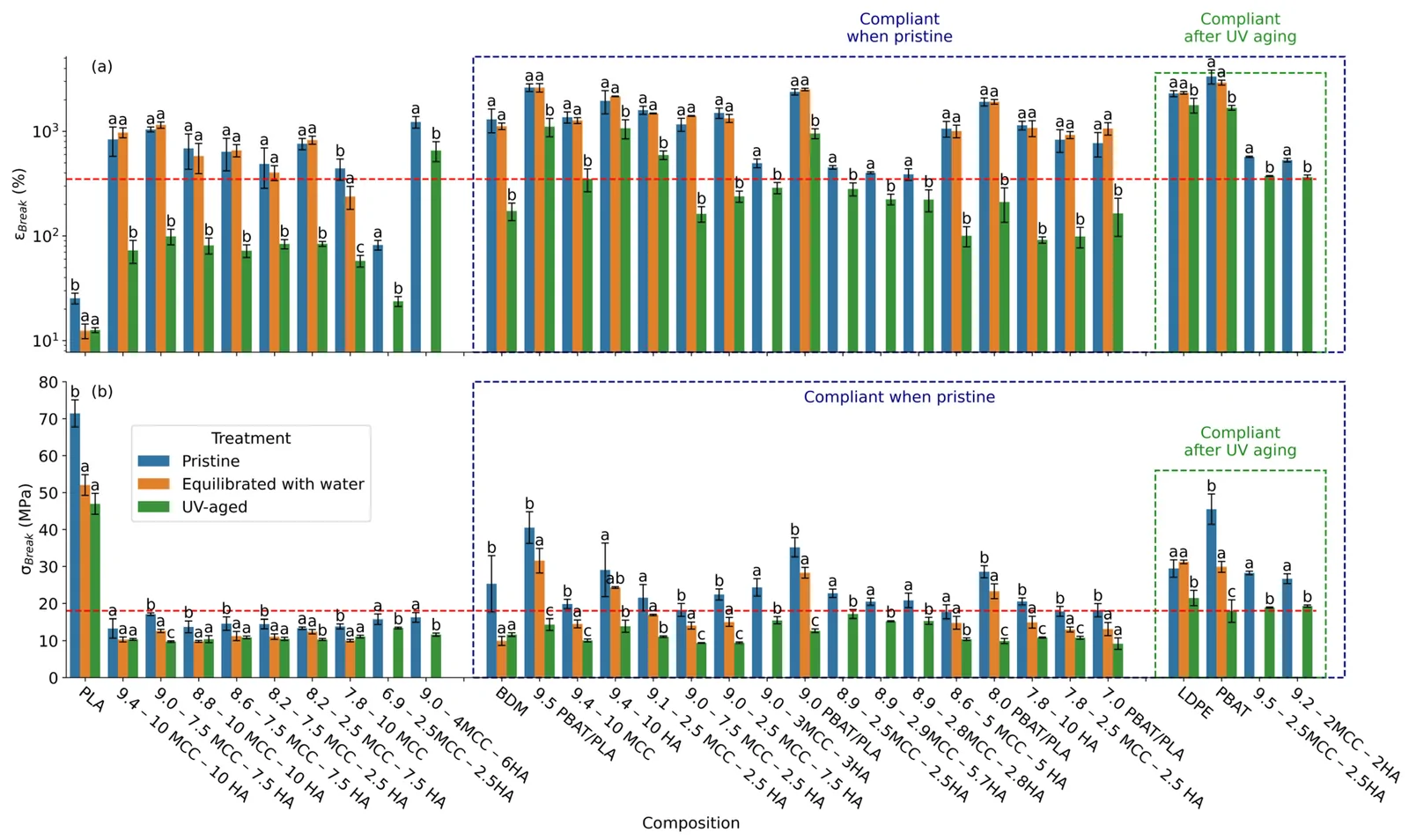
Mechanical properties (mean ± standard deviation, *n* = 3) for all samples before (pristine) and after aging (UV-accelerated aging or H_2_O saturation). Mechanical properties include (**a**) *ε_Break_* and (**b**) *σ_Break_*. Samples that were within error of satisfying EN 17033 criteria are outlined with boxes. The compact letter display labels (a, b, c) indicate the results of Tukey’s HSD post hoc test comparing the pristine, UV-aged, and H_2_O-saturated treatments within each composition. Treatments sharing the same letter are not significantly different (*p* > 0.05), whereas treatments with different letters differ significantly (*p* < 0.05). The dashed red lines represent the EN 17033 criteria for pristine BDMs: ≥350% and ≥18 MPa for *ε_Break_* and *σ_Break_*, respectively. Full stress–strain curves from which mechanical properties were calculated for all samples and treatments are provided in Supporting Information.

Mechanical properties are critical to agricultural mulch applications and are the first step in identifying viable compositions suitable for BDM films [8,18]. The ideal BDM film is durable during the growing season and degrades within 24 months after being tilled into the soil [70,71]. Pristine PLA exhibited a high *σ_Break_* (~71 MPa) but limited *ε_Break_* (~25%), which confirms its brittle nature (Figure 1) [8]. Pristine PBAT displayed a lower *σ_Break_* (~45 MPa) but extremely high ductility (~3300%), consistent with the literature (Figure 1) [8]. The PLA/PBAT blend exhibited intermediate mechanical behavior. Increasing the PBAT concentration increased *ε_Break_* while reducing *σ_Break_* (Figure 1). For example, the 5/95 PLA/PBAT exhibited ~40 MPa *σ_Break_* and ~2616% *ε_Break_*, whereas 20/80 PLA/PBAT exhibited ~29 MPa *σ_Break_* and ~1912% *ε_Break_* (Figure 1). The addition of 10 wt% MCC significantly reduced *ε_Break_*, with most samples exhibiting values below 500%. HA was less mechanically disruptive at equivalent loadings (Figure 1). Twenty-one compositions satisfied EN 17033 (within error) criteria (≥350% and ≥18 MPa for *ε_Break_* and *σ_Break_*, respectively) in the pristine state before aging (Figure 1). In general, compositions that satisfied the EN 17033 mechanical requirements exhibited a PBAT:PLA ratio of approximately 9:1 (Figure 1).

EN 17033 specifies a minimum initial *σ_Break_* and *ε_Break_* of 18 MPa and 350%, respectively, but does not define corresponding requirements or acceptable property retention after environmental aging [18]. Therefore, these initial-property thresholds were also applied after UV exposure as conservative design criteria for comparing formulation durability. Only four formulations retained both a *σ_Break_* of at least 18 MPa and an *ε_Break_* of at least 350% after UV exposure (Figure 1). This result indicates that resistance to UV-induced mechanical deterioration, rather than initial mechanical performance alone, was the limiting factor under this conservative assessment.

Mulch degradation is bifurcated into abiotic and biotic degradation mechanisms [70,71]. Abiotic degradation primarily occurs due to solar irradiation when the film is applied on the soil surface [70,71]. Biotic degradation dominates after tillage into the soil, where solar irradiation is diminished [70,71]. In this work, we characterized abiotic degradation due to solar irradiation (i.e., UV irradiation) and water absorption. Mechanical testing revealed a significant reduction in mechanical performance due to UV aging but not water absorption (Figure 1). However, the different durations of exposure and temperatures limit comparison. For example, water absorption required 80 days, while UV aging involved simulating 90 days of UV exposure. UV irradiation resulted in significant embrittlement across all compositions, which is consistent with the literature [72]. Our samples exhibited a larger decrease in mechanical performance compared to those of Rodriguez et al. [8], likely due to the aging schedule with more frequent temperature cycling (8 h on, 8 h off in Rodriguez et al. [8] versus 8 h on, 4 h off used in this work). Temperature is a critical parameter in the abiotic degradation of BDMs, which is why BDM durability is location-specific [73]. The magnitude of ductility loss increased with increasing PLA concentrations; 20/80 PLA/PBAT exhibited an approximately 90% reduction in ε_Break_ after aging. σ_Break_ reductions were also observed, particularly in PBAT-rich systems (Figure 1).

Most properties, including water absorption due to the presence of hydrophilic end groups, will be affected by a material’s aging history [74]. Commercial PBAT/PLA BDM became weaker, more hydrophilic and more brittle after exposure to plasma [75]. A three-month burying of film in soil resulted in 1–3 µm cracks, while an untreated sample showed no signs of degradation [75]. The hydrophilicity of the film improved the cell adhesion of *Pseudomonas guariconesis*, which is a critical factor in biofilm colonization [75]. Vasylius et al. [72] evaluated the mechanical properties of LDPE after UV aging with a 2.45 W·m^−2^ UVA-351 fluorescence lamp for 1–40 h at 60 °C. They found that LDPE brittleness increased with as little as 1 h of UV exposure [72]. Interestingly, *σ_Break_* increased with UV exposure, but ductility was more susceptible than strength to UV aging [72]. Dil et al. [76] showed that *ε_Break_* and impact strength were enhanced with 3 wt% silica for 80/20 and 70/30 PLA/PBAT. Ma et al. [77] investigated PBAT/PLA/CaCO_3_ blown film BDMs with 10, 20, and 30 wt% CaCO_3_ and demonstrated that both filler concentration and particle size distribution (PSD) significantly influence film performance. Increasing CaCO_3_ loading generally increased the storage modulus, complex viscosity, and oxygen and water vapor barrier properties, while the optimum overall mechanical performance (tensile strength, elongation at break, and tear resistance) was achieved using approximately 5 μm (compared to 0.4, 12, and 30 μm) CaCO_3_ due to its uniform dispersion, effective stress transfer, and lack of agglomeration [77].

Evaluating abiotic degradation is critical for maintaining mulch film performance throughout the intended growing season, whereas biotic degradation is essential for achieving sustainability goals after the film’s service life. In accordance with EN 17033 [18], BDMs must retain sufficient mechanical performance during field use while ultimately undergoing mineralization following incorporation into the soil. The standard defines biodegradation as the microbial mineralization of organic carbon in the film to carbon dioxide, water, mineral salts, and biomass, requiring at least 90% mineralization within 24 months under controlled aerobic soil conditions relative to a reference material [18]. Once the mulch film is tilled into the soil, biotic degradation becomes the dominant degradation pathway, with hydrolysis and microbial activity within the soil microfauna, microbiome, and rhizosphere playing a greater role than photodegradation [27]. Consequently, understanding the thermal stability and degradation behavior of the films is essential for interpreting their durability during use and their potential to undergo subsequent biodegradation following incorporation into soil.

### 3.2. Thermal Properties

**Figure 2:**
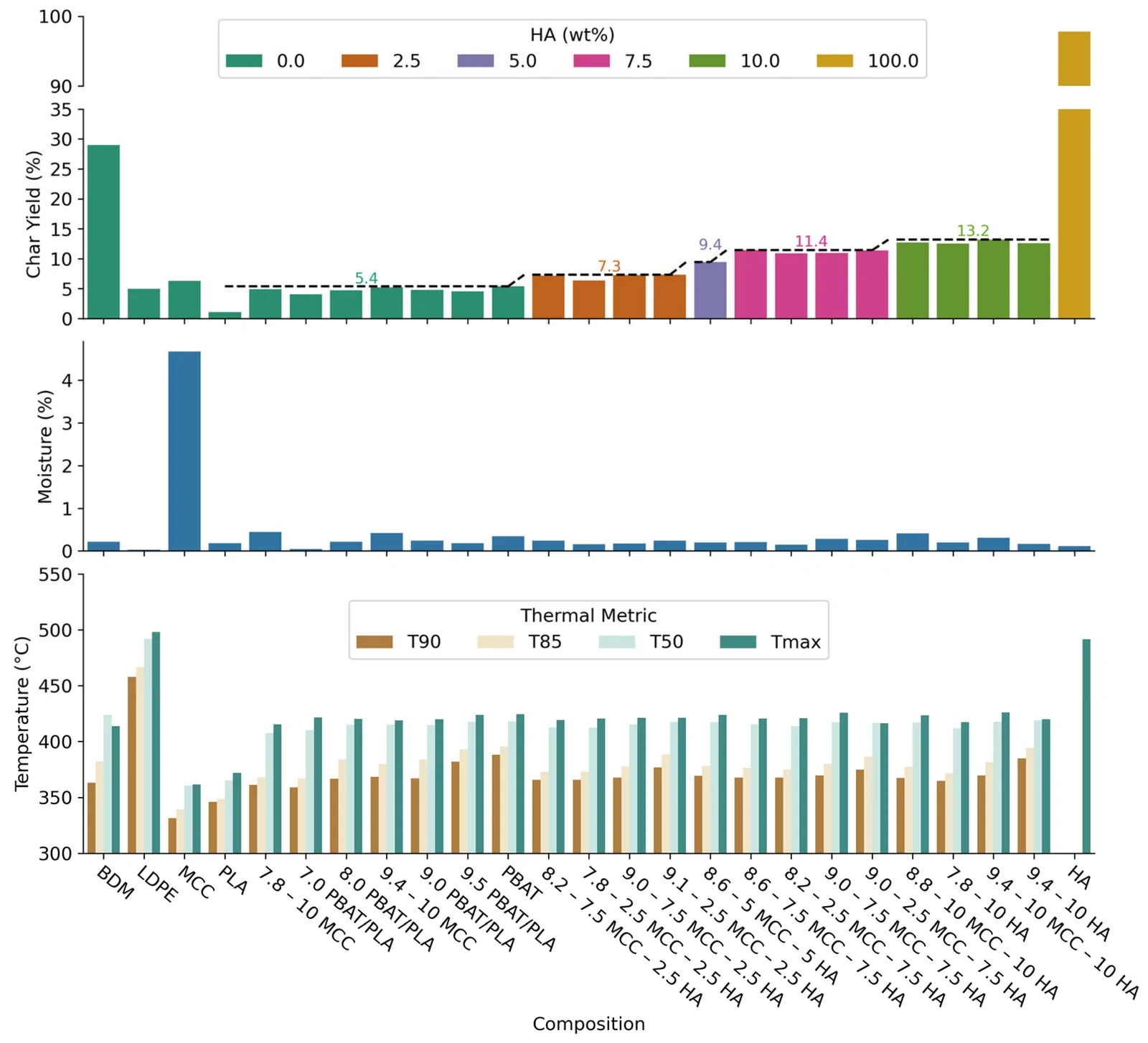
Char yield (%), moisture (%), and degradation parameters (T_90_, T_85_, T_50_, and T_Max_) (°C) for each composition, determined using TGA. Bars are colored according to the sample’s HA concentration.

Thermal properties serve as indicators of composition and thermal stability. Thermal degradation behavior is governed by the balance between organic matrix decomposition and the stabilizing contribution of inorganic residue [78]. The thermal stability of the PLA/PBAT-based systems is strongly influenced by matrix composition and filler incorporation (particularly HA) (Figure 2). The full TGA traces from which thermal parameters (char yield, moisture, T_90_, T_85_, T_50_, and T_Max_) are available can be found in the Supporting Information.

The lower decomposition temperature of PLA is attributed to its susceptibility to intramolecular transesterification, backbiting, and depolymerization to lactide, whereas the aromatic terephthalate segments in PBAT increase the activation energy for thermal degradation and delay mass loss [79]. Blended systems exhibited intermediate degradation behavior, which indicates limited synergistic stabilization between the two polymer phases. As an inorganic mineral phase, HA does not undergo phase transformation below 600 °C (see Supporting Information) and contributes directly to char yield (Figure 2) [80]. For this reason, formulations with HA demonstrated increased residual mass at elevated temperatures (Figure 2). The char yield we obtained for pure HA, 97.8%, was slightly greater than that of Wang et al. [80], at 96% (also at 10 °C·min^−1^); this outcome is potentially due to our long-term storage under nitrogen that prevents exposure to atmospheric water. TGA traces also revealed a greater moisture concentration for MCC at 4.7%, consistent with others (e.g., [81]) under similar humidity conditions (Figure 2, see Supporting Information for full TGA trace). Although MCC was stored under nitrogen, its hydrophilicity still allows for moisture retention during short periods of time of handling under ambient conditions. The thermal properties of MCC were also characterized using DSC (see Supporting Information). The first and second heating curves reveal a singular endothermic decomposition phenomenon in the heating curve instead of reversible melting (see Supporting Information). Overall, these results support that MCC exists as a particle in our composite as opposed to melting with PLA and PBAT during processing. These results were also reproduced at a slower heating rate of 5 °C·min^−1^. The observed moisture retention of MCC (Figure 2) further suggests that filler composition may influence water uptake in the composite films, which demonstrates the importance of water absorption behavior.

### 3.3. Water Absorption

Water absorption characterization is critical, as water is a key driver of both abiotic and biotic degradation [82]. The swelling effect and mechanical stress induced by water retention also facilitate biodegradation by allowing the diffusion of enzymes [83]. As degradation occurs, the interactions between cellulose and the hydrophobic polymer matrix are only expected to increase because the esters will be converted to carboxylic acid end groups during chain scission [84]. Therefore, the polyesters will serve not only as hydrogen bond acceptors but also as hydrogen bond donors and increase hydrophilic interactions [84,85]. Water absorption < 1% is characteristic of hydrophobic polymers used in conventional plastic mulch [25]. Once tilled into soil, increasing BDM hydrophilicity will enhance biodegradation by promoting water uptake and accelerating hydrolytic chain scission, which lowers the molecular weight and increases susceptibility to subsequent enzymatic/microbial assimilation [82]. On the other hand, film hydrophobicity is critical to ensuring their durability over the course of the growing season by preventing hydrolysis (and biodegradation via enzymatic degradation) [25]. Water absorption is also pH-dependent and, therefore, the soil pH will affect the swelling of the composite once tilled into the soil [86]. Acidic (pH < 7.4) or basic (pH > 7.4) conditions are known to expedite hydrolytic degradation via carbonyl protonation (and then H_2_O attack) or hydroxide attack, respectively [87]. Water absorption in the film is critical to the film’s integrity.

**Figure 3:**
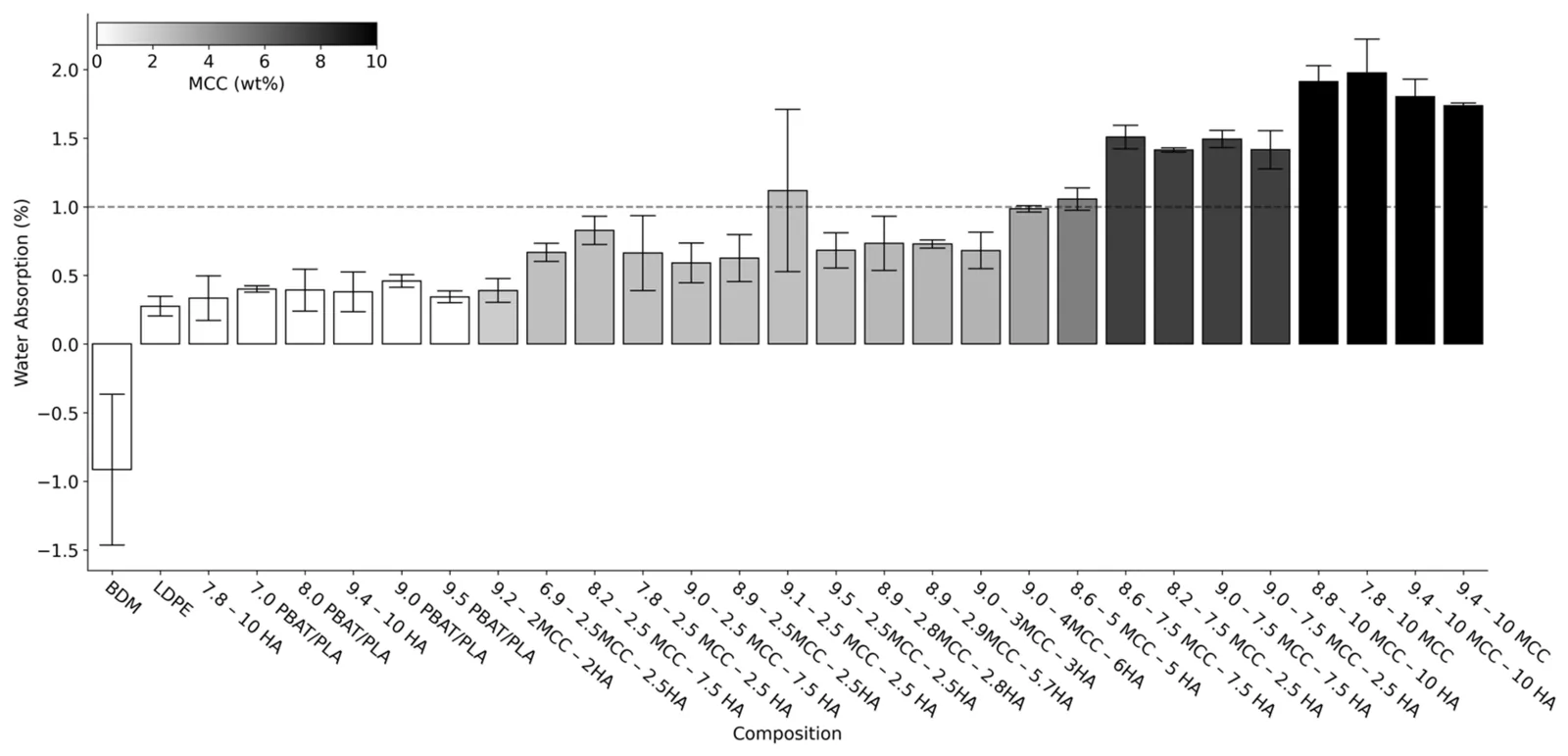
Average ± standard deviation (*n* = 3) water absorption at saturation (equilibrium) for each composition. The gray dashed line at 1% represents the typical upper limit for hydrophobic polymers. Bars are colored according to their MCC concentration.

Our composites consist of a PLA/PBAT hydrophobic polymer backbone and MCC/HA particle additives (see Supporting Information for a diagram). MCC exists as particles, as evidenced by DSC results that do not show melting prior to thermal degradation (see Supporting Information). Water absorption in the composites ranged from 0.33% (20/70/10 PLA/PBAT/HA) to 1.98% (20/70/10 PLA/PBAT/MCC) (Figure 3). Filler-free PLA/PBAT blends exhibited low equilibrium water uptake, ranging from approximately 0.34% to 0.39%, regardless of PLA concentration (Figure 3). By contrast, systems containing 10 wt% MCC demonstrated substantially greater water absorption values (1.74–1.98%) (Figure 3). Water absorption results reveal that LDPE and the polyesters in the hydrophobic backbone of our experimental samples do not contribute significantly to hydrophilic hydrogen bonding interactions because there is little uptake (Figure 3). The results from studying water absorption in LDPE, PLA/PBAT, and BDM (Figure 3) were consistent with those in the literature [8].

In our work, MCC concentrations revealed the greatest impact on water absorption (Figure 3). Overall, hydrophilic interactions with MCC were expected due to its rich hydroxyl concentration. Sambudi et al. [86] documented a similar phenomenon with their composite of orange peel powder (containing cellulose) and Ingeo^TM^ 3251D PLA. The increased water uptake of composites with MCC is also corroborated by its greater moisture concentration under ambient conditions compared to all other materials used in this study (see Supporting Information). Cellulose chains form hydrogen bonds via their hydroxyl groups [85]. Although we could not characterize the water absorption of MCC or HA powders using the same approach as the films, we measured the moisture concentrations of MCC and HA with TGA (Figure 2). In contrast to MCC, for the pH condition studied, the moisture concentration for HA was 0.111% and therefore did not exhibit significant hydrogen bonding. HA was, however, expected to contribute to water absorption based on studies with HA nanoparticles (NPs), where Yan et al. [88] reported as much as 2.2% water absorption with a formulation of 78.3/8.7/5/8 wt% PLA/PBAT/MCC/HA. Because the particles used in our study were larger than NPs, their effect may have been overshadowed by the presence of MCC.

The commercial BDM was the only composition in our experiments that lost weight during water absorption (Figure 3). Tan et al. [89] reported the disintegration of their BDM (poly(vinyl alcohol)/kenaf fiber/urea composite), where they found a 25% reduction in weight after 7 days of submersion. Film disintegration in water may be due to the high additive concentration, as evidenced by the char yield of 29% (Figure 2). Water uptake by hydrophilic particles causes mechanical stress in the form of hygroscopic swelling, which results in film deterioration [90]. The composition of this BDM was not made available to us due to being proprietary but was advertised as containing polyesters and starches. The moisture concentration of the commercial BDM (0.219%) was comparable to that of PLA/PBAT formulations, which ranged from 0.05 to 0.44% (see Supporting Information). While water absorption quantifies the extent of moisture uptake by the composite films, it does not directly reveal the chemical changes associated with abiotic degradation. Therefore, FTIR spectroscopy was performed to evaluate compositional changes in the polymer matrix and identify chemical modifications resulting from UV aging, which is a significant abiotic stress for films applied to fields [12].

### 3.4. FTIR

**Figure 4:**
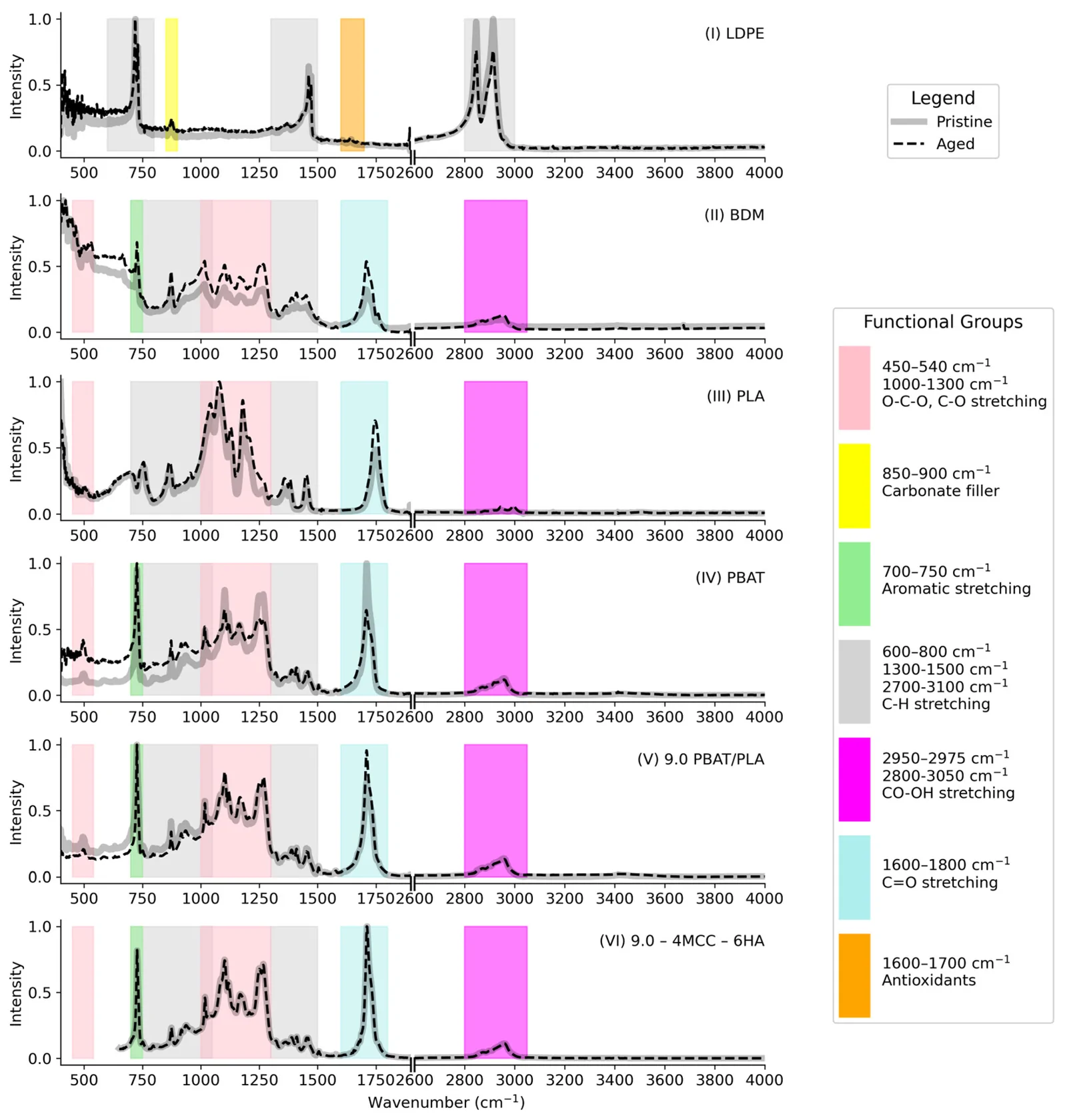
FTIR spectra key compositions following individual min–max normalization. Key functional group regions are color-coded to facilitate comparison of characteristic absorption bands independent of absolute absorbance. Formulation (VI) 9-4MCC-6HA is shorthand for 30^V^ (9.3 wt% PLA, 80.7 wt% PBAT, 3.5 wt% MCC, and 6.5 wt% HA).

FTIR analysis was conducted on pristine and UV-aged specimens to gain insight on polymer and additive chemistry, potential interactions, and aging progression. FTIR-ATR was selected over transmission FTIR due to its suitability for samples with varying thicknesses or ductility [91]. Overall, the spectra revealed peaks characteristic of polyesters, polyolefins, and mineral additives (Figure 4). The FTIR traces for the full experimental set are provided in the Supporting Information. The LDPE spectra included typical strong CH_2_ stretching peaks (asymmetric 2915 cm^−1^, symmetric 2847 cm^−1^), CH_2_ bending deformation peaks (1473 and 1462 cm^−1^), CH_2_ rocking deformations (725 and 691 cm^−1^), and additive signatures [92,93]. These additives likely involved CaCO_3_, which exhibits key peaks at 1391.24, 871.65, and 712.48 cm^−1^ [94].

CI and CAI were used as indicators of degradation, as the ester bond is where cleavage occurs and, therefore, changes in relative intensity and chemistry (i.e., the backbone ester becomes a carboxylic acid end group) [79,95]. The pristine (as purchased) LDPE used in our study had minor carbonyl formation, with an initial CI of 0.33 due to manufacturing and storage under ambient conditions (Figure 4) [19,96]. CIs have been reported to range from 0.32 to 0.47 for LDPE, 0.91 to 2.36 for PLA/PBAT, and 16.03 to 31.51 for PBAT-based commercial BDMs [19,96]. Jubinville et al. [97] used the carbonyl range of 1650 cm^−1^–1850 cm^−1^ with the CH_2_ methylene region of 1420 cm^−1^–1500 cm^−1^ while assessing the degradation of high-density poly(ethylene) (HDPE) during recycling; our range is shifted slightly to 1384 cm^−1^ to 1472 cm^−1^ to capture our peaks. The peak ranges were customized to our samples because individual FTIR traces are sensitive to the equipment, sample preparation (in the case of transmission FTIR), sample thickness, crystallinity, molecular weight, and other properties inherent to different grades of the same polymer [91,96,98].

Our results indicate that mechanical testing revealed changes associated with UV aging that were not readily apparent by FTIR spectroscopy. UV aging produced relatively small, formulation-dependent changes in both the CI and CAI. While several formulations exhibited statistically significant differences, most showed no measurable change after aging (87% for CI, 85% for CAI), which indicates limited surface oxidation under the applied exposure conditions (see Supporting Information). Furthermore, significant changes in CI were not consistently accompanied by changes in CAI because the two indices probe different chemical features: CI reflects the total carbonyl concentration, including ester carbonyls already present in PLA and PBAT, whereas CAI is more sensitive to carboxylic acid groups formed during chain scission. These results demonstrate that FTIR responses to UV aging were modest and formulation-dependent, highlighting the complexity of degradation processes in biodegradable polyester blends. However, the absence of statistically significant changes in CI or CAI does not preclude degradation, as chemical changes during polymer degradation may be too subtle to produce measurable differences in FTIR indices. Ji et al. [99] found that a seemingly monomodal carbonyl peak centered at 1753 cm^−1^ consisted of two peaks at 1775 cm^−1^ (PLA carbonyl shifted to a higher energy due to hydrogen bonding) and 1751 cm^−1^ (amorphous PLA carbonyl that is not hydrogen-bonded) using peak deconvolution. FTIR chemical shifts for oxidation products can easily overlap [100]. Additionally, detecting oxidation using polymers with existing carbonyl groups (e.g., polyesters like PLA and PBAT) is less reliable than detection using polymers without those existing groups (e.g., LDPE) [101].

Consistent with this finding, previous studies investigating the abiotic and/or biotic degradation of LDPE, PLA, and PBAT using FTIR have reported variable outcomes, with the extent of detectable chemical changes depending on the polymer system and aging conditions (time, environment) [101,102]. Carbonyl groups are generated through oxidation while simultaneously being transformed, cleaved, leached, and biologically consumed during abiotic and biotic degradation [101,103]. Furthermore, there is also a lag period during which polymer molecular weights are too large for biotic degradation [104]. Li et al. [105] achieved biodegradation of PLA with an initial M_w_ of 170,822 g·mol^−1^, M_n_ of 90,537 g·mol^−1^, and PDI of 1.89. Nelson et al. [106] achieved biodegradation of PBAT with a starting M_w_ and M_n_ between 11,700 and 17,150 g·mol^−1^ and between 37,260 and 52,800 g·mol^−1^, respectively. The exact initial molecular weight needed for degradation in soil is not known. Moreover, overlapping intrinsic carbonyl signals, surface topography, biofilms, and spectral processing techniques all influence CI [103]. Because ATR-FTIR only probes the top few micrometers of polymer samples [107], surface-associated biofilms made up of proteins, polysaccharides, and nucleic acids contribute substantially to the spectrum, which complicates the interpretation of polymer oxidation [103,108]. This limitation may explain why the literature on degradation in environments prone to biofilms (e.g., marine environments, soil, and cultures) exhibits complex changes in FTIR spectra [108,109,110]. For example, Xiong et al. [96] did not observe a significant change in the CI of LDPE (6, 8, 10, and 15 µm thicknesses) after application for 1 year on a gray desert soil field at the Arid Ecosystem Experimental Station in Gansu Province, China. Instead, SEM images and mechanical testing revealed fragmentation and reduced strength/ductility after aging, especially for the thinnest film (6 µm) [96]. Similarly, Xu et al. [15] found visible degradation in their films before the effects were measurable in FTIR spectra. Zhang et al. [111] aged PE and BDM MPs in silty loam soil for a 255-day incubation period, then compared FTIR CI before and after. They found that CI decreased for composite BDMs and increased for PE and a PLA/PBAT blend (without additives) due to biotic aging in soil [111]. Graf et al. [19] evaluated the biodegradation of seven 10.5–29 μm thick BDMs comprised of biodegradable polyesters and additives over 12 months in North Wales, U.K., after being applied to the soil or buried as macroplastics. Regardless of composition, BDMs showed larger changes in CI when buried in soil as opposed to the abiotic (e.g., solar irradiation, precipitation, and mechanical stresses) damage sustained while above ground [19]. In summary, FTIR is a powerful tool for characterizing the chemical structure and functional groups of polymeric materials; however, degradation-induced changes may not always produce measurable spectral differences despite significant reductions in mechanical performance. To further investigate changes in the bulk structure associated with UV aging, XRD was employed.

### 3.5. XRD

**Figure 5:**
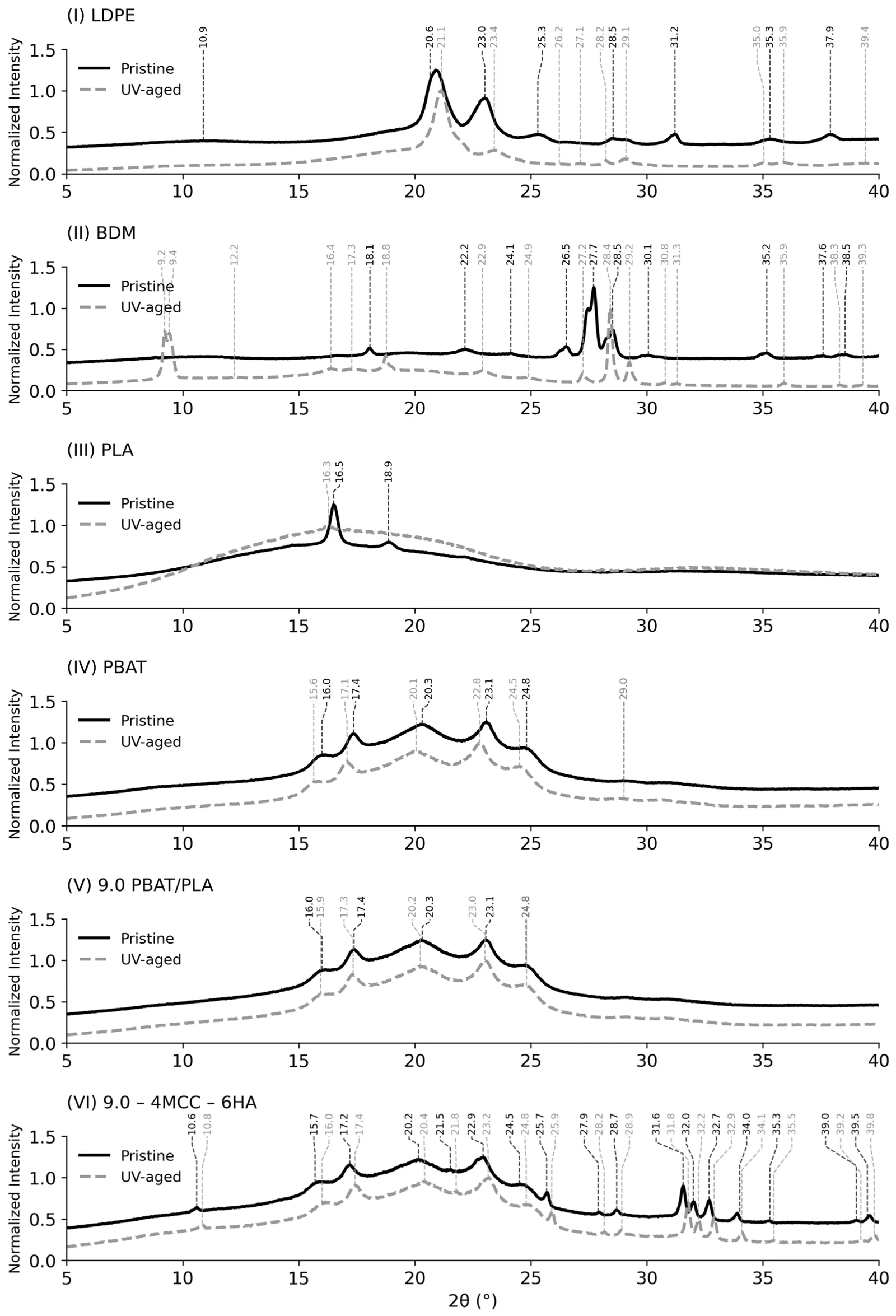
Representative XRD patterns of selected film compositions before and after UV aging. Diffraction intensities were normalized to the maximum intensity of each individual pattern to enable comparison of peak positions and relative peak shapes independent of absolute diffraction intensity. Formulation (VI) 9-4MCC-6HA is shorthand for 30^V^ (9.3 wt% PLA, 80.7 wt% PBAT, 3.5 wt% MCC, and 6.5 wt% HA).

XRD was used to assess crystallinity for the PLA/PBAT grades used in this work. XRD was selected over DSC because overlap among PBAT melting, PLA cold crystallization, and PLA melting complicates the separation of their thermal contributions and reliable calculation of component-specific crystallinity [8,112]. XRD analysis revealed modest crystallinity across most experimental blends (approximately 3–7%), with the greatest contributions originating from HA (Figure 5). The XRD patterns revealed signature peaks for each constituent that allowed for the calculation of crystalline concentrations and crystallite size (see Supporting Information).

The HA diffractogram revealed a large array of peaks consistent with its mineralogy [113]. PLA, PBAT, MCC, and LDPE are semicrystalline polymeric materials and therefore exhibited less well-defined crystalline peaks with large amorphous halos (Figure 5); the diffractograms are consistent with those in other studies [45,112,114,115].

The commercial BDM reference exhibited a substantially greater *χ* (~19.5%), indicating a fundamentally different structure compared to the pristine experimental blends, which ranged from 3.5 to 8.1%. Crystallite size variations in formulations without HA ranged between 28.9 nm and 209.0 nm, while adding HA varied the crystallite size between 71.3 nm and 169.5 nm (Figure 1). The average crystallite size was 126.34 nm when HA was in the formulation and 73.5 nm for formulations without HA. When considering peaks due to PLA, PBAT, or MCC (within the range of 15–25°), the differences in crystallite size reduce significantly. For example, for peaks within this range, the average crystallite size for formulations with and without HA are 47.2 and 43.6 nm, respectively (Figure 1 and see Supporting Information). Therefore, crystallite size reflects more the HA concentration and alone does not explain the observed differences in mechanical behavior among the formulations. The XRD traces for the full experimental dataset and a table summarizing all properties are provided in the Supporting Information. Crystallinity may increase or decrease during UV-induced degradation: preferential degradation of amorphous regions [116] and chain-scission-induced crystallization increase crystallinity [117], whereas damage to crystalline regions decreases it [118]. In these composites, overlapping diffraction peaks from PLA, PBAT, MCC, and HA also complicate interpretation (Figure 5). Crystallinity alone, therefore, cannot reliably indicate the extent of degradation, which was interpreted alongside the mechanical and chemical characterization.

In our work, we saw a decrease in crystallinity after LDPE UV aging from 27.2% to 18.2%, while for our experimental samples, the changes were highly varied (Figure 5 and see Supporting Information). Crystallinity ranged from 3.48% to 8.96% in pristine experimental formulations and from 0.05% to 9.08% after aging (Figure 5 and Supporting Information). Across the experimental formulations, UV aging produced changes in crystallinity ranging from −3.96% to +4.30% (Figure 5 and Supporting Information). In general, samples with more HA had higher crystallinity regardless of whether they were pristine or aged. Interestingly, the crystallinity of pure PLA decreased dramatically (from 4.00% to 0.05%) after UV aging, as evidenced by the relatively larger amorphous halo in the XRD diffractogram after aging (Figure 5). The variable responses are evidence of the competing processes of chemicrystallization, the formation of new crystalline regions following degradation-induced chain scission, and the degradation of both amorphous and crystalline regions [119]. For example, Najmeddine et al. [116] observed an increase in LDPE crystallinity from 43% to 52% after aging it for 112 h under a 254 nm lamp. Zhang et al. [120] observed a decrease in LDPE crystallinity after 2 weeks of UV aging (340 nm aging chamber, 0.51 W·m^−2^·nm^−1^), followed by an increase after 3 weeks under the same conditions, relative to the pristine material. Li et al. [105] observed a decrease in crystallinity during the biodegradation of PLA by lipase and protease enzymes produced by *Bacillus safensis*. These results suggest that competing degradation and chemicrystallization processes result in UV-dose-dependent outcomes [120].

### 3.6. SEM

SEM was primarily performed to obtain insight into the PSDs of the powdered constituents and to characterize the morphology of MCC and HA particles. (SEM images of MCC and HA particles are provided in the Supporting Information.) SEM with energy-dispersive analysis was initially explored as a potential method for the elemental analysis of the film surfaces, with the intent of distinguishing the heavier elements in HA from the polymeric components (PLA, PBAT, and MCC). However, SEM analysis (with a conductive coating) of the composite films beyond surface morphology was not feasible due to severe sample damage incurred during electron beam scanning (images available in Supporting Information). The films were highly susceptible to beam-induced deformation, and no imaging conditions provided sufficient resolution without simultaneously damaging the sample. Additionally, SEM imaging of a representative composite film did not reveal significant indicators of UV aging, and, therefore, SEM analysis was not continued for additional film samples. Although SEM was not suitable for assessing the degradation or elemental analysis of the films, it was effective for the characterization of the powdered constituents and for obtaining PSDs that contextualized additional material properties. MCC and HA PSDs, which are provided in the Supporting Information, demonstrated substantial differences in particle size and distribution. HA exhibited a narrower PSD with a mode centered at approximately 12–15 μm, which indicates a relatively uniform population of smaller spherical particles. By contrast, MCC displayed a broader distribution with a primary mode near 35–45 μm and a secondary shoulder extending toward approximately 80–120 μm, which suggests greater heterogeneity in particle size and morphology. This difference is consistent with the irregular fibrous structure of MCC compared to the comparatively spherical morphology of HA [121,122].

### 3.7. Regression

**Figure 6:**
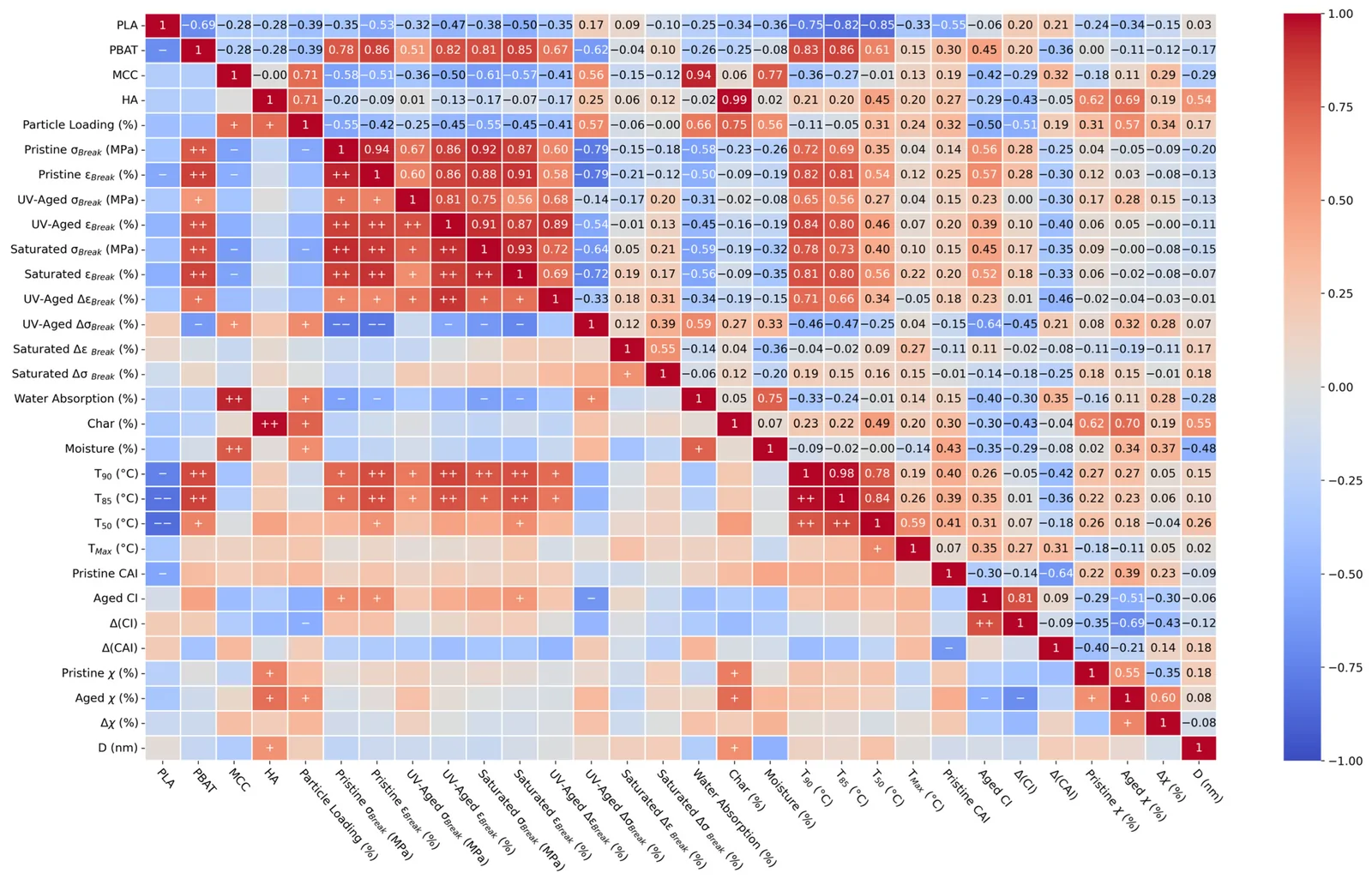
Heat map of correlation matrix among inputs and outputs for DoE dataset. + + indicates r > 0.75, + indicates 0.5 < r ≤ 0.75, − indicates −0.75 < r ≤ −0.5, and −− indicates r < −0.75. The equivalency diagonal and symmetrical upper portion of the heat map provides r values, while the lower triangle uses symbols to highlight significant effects. Data were limited to DoE dataset only.

Regression analyses examine composition–property relationships using measurements from distinct specimens of each formulation. A correlation matrix, or heat map, was used as a preliminary assessment of multicollinearity or any correlations among predictors and responses (Figure 6). The heat map is a representation of the Pearson correlation coefficient (r) between each pair of features and revealed some preliminary associations that guided more in-depth regression. Note that specimens shared melt-mixed batch processing and therefore were not replicates prepared from independent batches [59]. The batch-to-batch reproducibility of prepared films was demonstrated in previous studies [8,112]. PLA concentration showed the strongest negative associations with thermal stability metrics, particularly T_50_, T_85_, and T_90_ (Figure 2 and Figure 6), which indicates that increasing the PLA concentration generally reduced thermal resistance within this DoE space. PLA was also associated with reductions in ductility across the pristine, saturated, and UV-aged conditions, as reflected by consistently negative relationships with *ε_Break_*. More moderate negative associations were observed with crystallinity, moisture concentration, and *σ_Break_*. Interestingly, PLA concentration was not strongly correlated with *σ_Break_*, despite tensile strength typically increasing with PLA concentration in prior PLA/PBAT blend studies [8,112]. The weak correlation between PLA concentration and pristine tensile strength may partly reflect the investigated PLA range of 0–30 wt%, as restriction of the range attenuates observed correlations [123]. A simultaneous variation in MCC concentration may further obscure this relationship because MCC dispersion and filler–matrix adhesion influence stress transfer and tensile strength [124]. These factors provide plausible explanations for the observed correlation but were not independently isolated in this study. Overall, increasing PLA concentration in this design space was generally associated with reduced ductility, lower thermal stability, and modest decreases in tensile performance.

PBAT concentration was strongly associated with improved ductility and tensile performance across the pristine, UV-aged, and saturated conditions (Figure 6). The strongest positive relationships were observed with *ε_Break_* under all conditions, as well as with pristine and saturated *σ_Break_*, which indicates that increasing the PBAT concentration promoted both flexibility and tensile performance within this design space. PBAT also exhibited strong positive associations with thermal stability metrics, particularly T_85_ and T_90_, which suggests improved resistance to thermal degradation. More moderate positive relationships were observed with UV-aged tensile retention metrics, while weak negative relationships were observed with water absorption, char yield, crystallinity, and degradation indices. Mechanistically, PBAT contributes ductility to the blend system, whereas PLA and rigid fillers restrict chain mobility.

MCC concentration was strongly associated with increased water absorption and moisture uptake, which indicates the hydrophilic nature of MCC within the composite system (Figure 6). By contrast, MCC concentration was generally associated with reductions in tensile performance, including decreases in both *σ_Break_* and *ε_Break_* under the pristine, saturated, and UV-aged conditions. Negative associations were also observed with several thermal stability metrics, although these relationships were comparatively weaker. For the UV-aged samples, more moderate relationships were observed with tensile changes, which suggests that MCC may influence the degradation of mechanical properties. Overall, these findings indicate that increasing the MCC concentration primarily promoted moisture uptake while reducing ductility and tensile performance within the investigated design space. HA concentration was primarily associated with increased char yield, crystallinity, crystallite size, and thermal stability, which indicates that HA mainly influenced the structural and thermal behavior of the composites (Figure 6). By contrast, HA showed only weak relationships with tensile properties and moisture uptake.

**Figure 7:**
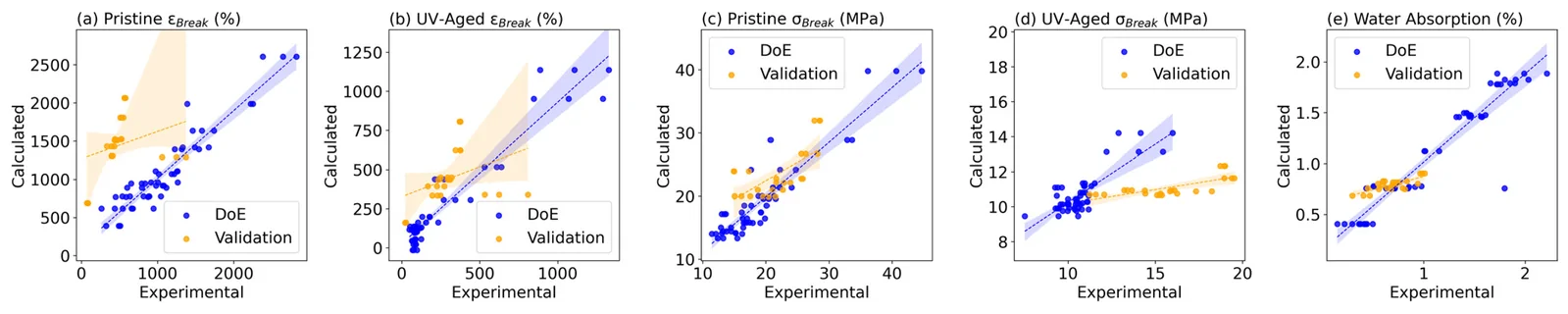
Predicted values for mechanical properties (*ε_Break_* and *σ_Break_*, before and after UV aging) and water absorption using Elastic Net regression. Predictions for (**a**) pristine *ε_Break_*, (**b**) UV-aged *ε_Break_*, (**c**) pristine *σ_Break_*, (**d**) UV-aged *σ_Break_*, and (**e**) H_2_O absorption. Shaded regions indicate 95% confidence bands.

Overall, the correlation patterns suggest that PBAT primarily contributed to improved mechanical performance, MCC strongly influenced water uptake behavior, HA dominated char formation and *χ* responses, and PLA concentration was generally associated with reductions in ductility and several thermal stability metrics. While these correlations provide useful insight into formulation–property relationships, a multi-variable regression model was used to analyze the relationships between inputs and outcomes and to develop a predictive tool to optimize desirable properties. An Elastic Net model was selected for regression because it was less sensitive to multicollinearity than non-regularized linear models (e.g., ordinary least squares). Multicollinearity was inherent in our design due to the mixture constraint in the composition. Furthermore, Elastic Net regression provides interpretable coefficients with mechanistic insight and does not require parametric datasets for usage. Concentration-based modeling for mechanical properties was overall less successful than that for strictly concentration-based properties like water absorption (Figure 7 and Table 5). This outcome is likely because mechanical properties are highly dependent on processing and morphology, which is not captured in composition inputs [112]. Predictive performance metrics (*R*^2^ and *nRMSE*) are summarized in Table 5. All responses exhibited strong agreement between the predicted and experimental values for the DoE dataset and relatively weak fitting on the validation dataset (Table 5). The weaker validation performance indicates limited model generalizability beyond the DoE training set. Elastic Net regression models were trained using the design of experiments dataset and evaluated using an independent validation dataset (Table 5). Regularization reduced the number of active predictors from the original 10 mixture terms to between three and six terms depending on the response, which indicates that only a subset of formulation variables was required to capture most of the variance in the dataset (Table 5).

Positive coefficients indicate an increase in the predicted response with increasing component fraction, whereas negative coefficients indicate a decreasing effect. Elastic Net regression models were fit with an intercept term. Although mixture compositions sum to a constant (e.g., PLA + PBAT + MCC + HA = 100 wt%) and a hypothetical formulation containing none of the components would have a zero response, forcing the intercept to zero distorted the coefficient estimates. In mixture systems, increasing the fraction of one component necessarily decreases the fraction of at least one other component with a constant sum constraint. Therefore, any predicted property change inherently reflects compositional changes rather than the effect of one ingredient alone (as implied by the presence of a single coefficient). In summary, individual coefficients cannot be interpreted as independent component effects [125]. If the intercept is constrained to zero, the model must attribute the entire baseline response to the predictor terms, which can force the regression to compensate by assigning uniformly negative or otherwise counterintuitive coefficients to correlated variables. Including an intercept allowed the model to capture the baseline response independently of compositional trade-offs, resulting in more stable and interpretable coefficients.

Model performance varied among responses and diverged particularly between the training and validation datasets (Table 5). The water absorption model exhibited the strongest predictive performance, with a training *R*^2^ of 0.893 and a validation *RMSE* of 0.18. This strong performance is consistent with the correlation analysis, where MCC showed strong positive correlations with water absorption (*r* = 0.94) and moisture concentration (*r* = 0.77) (Figure 6). Because water absorption is largely controlled by a single compositional factor, the regression model was able to capture the relationship with relatively few active terms. These results indicate that moisture is not strongly morphology-dependent (e.g., *χ*) in this system. The intrinsic hydrophilicity of the individual components governs water uptake rather than morphological effects (e.g., crystallinity) [87,126]. For example, water diffusion is highly dependent on polymer crystallinity, with amorphous regions being more permeable to water than crystalline domains [87]. Lendvai et al. [126] found that the greater D-isomer concentration of Ingeo^TM^ 2003D PLA (~4% D-isomer, water absorption 0.71%) resulted in greater water absorption compared to the Ingeo^TM^ 4032D (~2% D-isomer, water absorption 0.3%) used in this work. The lower D-isomer concentration of Ingeo^TM^ 4032D results in greater crystalline and mesophase concentrations compared to Ingeo^TM^ 2003D [127], which would explain the lower water retention.

The negative validation values observed for four of the five responses (Table 5) indicate that these models predicted the validation data less accurately than the training-set mean, despite *nRMSE* values that were comparable to those of the DoE dataset for some responses (e.g., water absorption). This apparent discrepancy is primarily due to the construction of the validation dataset. Validation formulations were manually selected to target compositions near the expected optimum, which resulted in a much narrower compositional range than the DoE dataset. For example, MCC concentration, the dominant predictor of water absorption with a standardized coefficient of 0.867 (Table 5), varied from 0 to 10 wt% in the DoE dataset but only from 2 to 3.53 wt% in the validation dataset. Consequently, the response variance of the validation set was substantially reduced, which made the R^2^, which is normalized by the variance of the observed data, sensitive to prediction errors.

Convex hull analysis showed that two of the eight validation formulations (23^V^ and 29^V^) were located outside the convex hull of the training dataset (see Supporting Information), indicating that predictions for these formulations represented extrapolation rather than interpolation. Still, inadequate validation performance cannot be attributed solely to extrapolation beyond the experimental design space. The negative validation values primarily reflect the limited generalizability of composition-only models rather than widespread extrapolation. Although the Elastic Net models successfully identified compositional trends and guided optimization toward regions of high desirability, the results indicate that mechanical performance is also influenced by non-compositional factors, such as processing-induced morphology, crystallinity, polymer orientation, and filler dispersion, which were not explicitly included in the model. These findings suggest that the models are better suited for identifying compositional trends and guiding formulation optimization than for accurately predicting the properties of previously unseen formulations.

The pristine *ε_Break_* model achieved a training *R*^2^ of 0.853 but performed poorly on the validation dataset (*R*^2^ = −55.8). Similarly, the UV-aged *ε_Break_* model showed moderate training performance (*R*^2^ = 0.836) but exhibited limited predictive accuracy for the validation samples (*R*^2^ = −4.85). These discrepancies suggest that ductility depends not only on composition but also on microstructural factors such as crystallinity, filler dispersion, and interfacial adhesion, which are not directly represented by the compositional predictors.

The pristine *σ_Break_* model showed moderate predictive performance, with a training *R*^2^ of 0.755 and a positive validation *R*^2^ of 0.665. This result indicates that tensile strength was more consistently related to formulation variables than elongation. By contrast, the UV-aged *σ_Break_* model exhibited lower predictive accuracy (R^2^ = 0.542 for training and R^2^ = −5.58 for validation), which suggests that UV-induced degradation introduces additional variability that is not fully captured by the compositional model.

Jia et al. [128] demonstrated that well-dispersed nano-CaCO_3_ at low wt% (typically 1–5 wt%) can improve mechanical properties, while larger loadings compromise properties. In our case, particle loading was negatively correlated with *σ_Break_* (Figure 6), and while MCC was negatively correlated with *σ_Break_* and *ε_Break_*, HA was not. This outcome is potentially due to MCC’s increased water retention (which would result in hygroscopic swelling) and larger particle size compared to HA. Differences in film thickness could also have affected properties because mechanical properties are thickness- and geometry-dependent [8,129]. Overall, in our work, the experimental samples (~330 μm thick) were the thickest, followed by commercial LDPE (76.2 μm thick) and commercial BDM (18.3 μm thick). Dog bone geometry was maintained constant for all samples using cutting dies, but control of sample thicknesses was limited to the film thicknesses commercially available. Sample degradation is also thickness-dependent [129]; mulch films, in practice, will be thinner (typically 10–80 μm but up to 150 μm for specialty crops) than the 330 μm samples used in our experiments [26]. Xiong et al. [96] also showed that mechanical integrity and durability were thickness-dependent for LDPE. Similarly, thicker BDMs have shown better retention of mechanical properties after field use [130]. For example, Cao et al. [131] found that thinner LDPE mulch films (5 μm) underwent faster oxidation with earlier fragmentation than thicker films (10 and 14 μm) during field aging, highlighting the importance of durability as a function of film thickness. Thickness also influences moisture uptake: thinner films reach moisture equilibrium sooner under comparable Fickian diffusion conditions [132]. Translation of the 9.3/80.7/3.5/6.5 wt% PLA/PBAT/MCC/HA formulation into thin blown films requires control of filler dispersion, moisture, thermal history, and blowing conditions, which influence film transparency, degradation, and mechanical properties [133,134]. The models were intentionally limited to composition-based predictors to isolate formulation–property relationships. Processing temperature was held constant, and mixing time ranged from 4 to 9 min until torque stabilized under the same processing protocol rather than being systematically varied. The film thickness of the samples exhibited incidental variations arising from processing but was not independently controlled or systematically varied as an experimental factor and was therefore excluded to maintain the composition-focused modeling approach. Mechanical properties in both machine and transverse directions, water-uptake kinetics, and UV-aging affects need to be assessed at the application thickness to determine whether further formulation or processing optimization is necessary.

The desirability optimization (Equations (3)–(5)) identified a composition containing 9.3 wt % PLA, 80.7 wt % PBAT, 3.5 wt % MCC, and 6.5 wt % HA. This formulation achieved a pristine elongation at break of 1227.1 ± 156.4%, UV-aged elongation at break of 654.8 ± 140.7%, pristine tensile strength of 16.2 ± 1.25 MPa, UV-aged tensile strength of 11.6 ± 0.4 MPa, and water absorption of 0.99 ± 0.02%. The PBAT-rich composition is consistent with the strong positive correlations observed between PBAT concentration and both elongation at break and tensile strength. Conversely, the relatively low PLA concentration reflects the reduction in ductility associated with increasing PLA concentration. As a result, optimization favored a highly flexible PBAT-dominated formulation while retaining sufficient PLA to maintain film integrity. Particle loadings were limited to 10 wt %, as greater solid concentrations could not be consistently processed with extrusion equipment, which constrained the experimental design space. Within these processing limitations, optimization favored a greater proportion of HA than MCC, which suggests that HA provided a more favorable balance between functionality and performance. The optimized composition satisfied EN 17033 requirements. The DoE identified an optimal PBAT/PLA ratio that consistently met the standard prior to aging and provides a robust compositional foundation for future formulation development. This optimized polymer ratio provides a baseline from which properties can be systematically tailored through the incorporation of functional particles, masterbatches, additives, and processing modifications. MCC and HA were incorporated as functional fillers relevant to the intended environmental performance of the films. Although MCC influenced water absorption and HA increased P concentration, these properties were not directly linked to abiotic or biotic degradation or fertilizer performance. Ultimately, establishing this composition-driven framework provides a pathway for the materials engineering of BDMs that satisfy agricultural performance requirements while advancing more sustainable crop production. The results indicate that post-aging performance cannot be explained by composition alone, underscoring the influence of non-compositional factors that warrant further investigation. These findings suggest that future optimization must expand beyond composition to identify and include non-compositional conditions that govern long-term durability.

## 4. Conclusions

In conclusion, BDMs with eco-friendly additives were achieved using a PLA/PBAT matrix with a PBAT/PLA ratio of nine. This study examined the influence of formulation composition on the film properties, performance, and behavior using correlation analysis and Elastic Net regression modeling. The results demonstrate that compositional variables strongly influence material performance, although some responses are also affected by microstructural factors not captured directly by formulation. Elastic Net regression reduced the number of active predictors while maintaining reasonable predictive performance for several responses. Interestingly, water absorption showed the most reliable predictive behavior, which reflects the dominant influence of MCC, whereas elongation models showed weaker validation performance, which suggests that ductility depends on additional structural factors such as phase morphology and filler dispersion. Overall, the results demonstrate that PBAT primarily governs ductility, MCC controls water uptake, and HA contributes to structural and thermal characteristics. Water absorption and HA concentration were characterized as formulation-dependent material properties relevant to the intended agricultural functions of the films. Future work will determine how these properties relate to abiotic and biotic degradation processes, P availability, and fertilizer performance in soil. Additionally, future work will incorporate additional characterization techniques and input parameters such as molecular weight via size exclusion chromatography.

The modeling approach provides useful insight into formulation–property relationships and offers guidance for designing PLA/PBAT-based composite films with targeted mechanical properties and controlled water absorption. Multi-objective optimization identified 9.3/80.7/3.5/6.5 wt% PLA/PBAT/MCC/HA as the preferred formulation within the tested design space. Decomposition due to UV aging was not quantifiable using FTIR or SEM but was detectable using tensile testing. The optimized formulation satisfied the tensile strength criterion of EN 17033. Although tensile strength decreased following accelerated UV aging, EN 17033 does not specify a requirement for aged films. The combined experimental and modeling approach used in this study therefore establishes a practical composition-screening framework and identifies the variables that should be added next: processing history, film thickness, morphology, field durability, and direct soil biodegradation.

## Figures and Tables

**Table 1:** Selected Properties of the Primary Polymer Matrices Used in This Work.

Property	PLA	PBAT	Ref.
Grade	Ingeo^TM^ 4032D	Ecoflex^®^ F Blend C1200	[41,42]
Manufacturer	NatureWorks LLC	BASF	[41,42]
Description	Semicrystalline, high-heat film grade	Flexible biodegradable film grade	[41,42]
Chemical Structure	Aliphatic polyester	Aliphatic–aromatic copolyester	[41,42]
Stereoisomerism	Predominantly poly(L-lactide) (PLLA) with small amounts of D-lactide stereoisomer units	Random copolymer of BA & BT units; no stereo-regular backbone configuration	[43,44]
Reported Composition	<5 wt%, ∼1.4% D-lactide	51.6/48.4 mol% BA/BT	[43,45]
M_n_ (g·mol^−1^)	107,296	36,500–52,100	[43,46,47]
M_w_ (g·mol^−1^)	183,177	67,000–104,200	[43,46,47]
PDI	1.71	1.29–2.85	[43,46,47]

M_n_: number-weighted average molecular weight; M_w_: weight-weighted average molecular weight; PDI: polydispersity index; BA: butylene adipate; BT: butylene terephthalate.

**Table 2:** Compositions of Experimental Formulations.

Run ID	PLA (wt%)	PBAT (wt%)	MCC (wt%)	HA (wt%)
1^D^	30	70	0	0
2^D^	21.25	73.75	2.5	2.5
3^D^	20	70	10	0
4^D^	20	70	0	10
5^D^	16.25	73.75	7.5	2.5
6^D^	16.25	73.75	2.5	7.5
7^D^	12.5	77.5	5	5
8^D^	11.25	73.75	7.5	7.5
9^D^	10	70	10	10
10^D^	8.75	81.25	7.5	2.5
11^D^	8.75	86.25	2.5	2.5
12^D^	8.75	81.25	2.5	7.5
13^D^	8.75	76.25	7.5	7.5
14^D^	5	75	10	10
15^D^	5	95	0	0
16^D^	5	85	0	10
17^D^	5	85	10	0
18^C^	100	0	0	0
19^C^	10	90	0	0
20^C^	0	100	0	0
21^C^	0	0	100	0
22^C^	0	0	0	100
23^V^	10.5	83.8	2.8	2.8
24^V^	10.3	81.1	2.9	5.7
25^V^	5	90	2.5	2.5
26^V^	8	88	2	2
27^V^	9	85	3	3
28^V^	10	85	2.5	2.5
29^V^	29	66	2.5	2.5
30^V^	9.32	80.68	3.53	6.47

The superscripts ^D^, ^C^, and ^V^ represent DoE, control, and validation datasets, respectively.

**Table 3:** Desirability Function Goals for Each Response.

Response	Goal Type	Lower Limit	Target	Weight
Pristine *ε_Break_* (%)	Maximize	350	420	1
UV-aged *ε_Break_* (%)	Maximize	350	420	1
Pristine *σ_Break_* (MPa)	Maximize	9	18	1
UV-aged *σ_Break_* (MPa)	Maximize	9	18	1
Water Absorption (%)	Maximize	0.8	1.0	1

**Table 4:** FTIR Integration Regions Used for Index Calculations for LDPE and Polyester-Based Composites.

Material	Functional Group		Reference Group
	C=O (cm^−1^)	COO-H (cm^−1^)	CH_2_ (cm^−1^)
LDPE	1536–1928	2950–2975	1384–1472
Polyester Composites	1552–1808	2800–3050	1336–1496

**Table 5:** Elastic Net Model Performance and Regression Coefficients.

Term	Pristine *ε_Break_*	UV-Aged *ε_Break_*	Pristine *σ_Break_*	UV-Aged *σ_Break_*	Water Absorption
Model Performance					
α	2.78 × 10^−2^	2.78 × 10^−2^	1.00 × 10^−1^	2.78 × 10^−2^	2.78 × 10^−2^
*l*_1_ ratio	1.000	1.000	0.611	1.000	1.000
Iterations	83	165	100	95	34
Active coefficients	4	6	3	5	3
DoE *R*^2^	0.853	0.836	0.755	0.542	0.893
DoE *RMSE*	219.67%	135.44%	3.46 MPa	1.01 MPa	0.194%
DoE *nRMSE*	8.6%	10.6%	10.4%	11.9%	9.4%
Validation *R*^2^	−55.775	−4.848	0.665	−5.578	−2.583
Validation *RMSE*	1140.58%	270.97%	2.36 MPa	5.27 MPa	0.183%
Validation *nRMSE*	225.2%	76.5%	17.3%	83.4%	53.6%
Regression Coefficients					
Response Unit	%	%	MPa	MPa	%
Intercept	−2449.24	453.68	−25.88	9.76	0.405
PBAT	52.08	7.46	0.623	4.35 × 10^−2^	–
MCC	–	–	−5.89 × 10^−2^	–	0.134
PLA × PBAT	−0.21	−0.39	–	−1.65 × 10^−3^	–
PLA × MCC	−0.81	0.18	–	1.59 × 10^−2^	7.07 × 10^−4^
PLA × HA	–	−1.91	–	−3.66 × 10^−4^	–
PBAT × MCC	−0.62	−0.73	−7.28 × 10^−3^	−4.48 × 10^−3^	–
MCC × HA	–	−0.98	–	–	1.19 × 10^−4^
Standardized Regression Coefficients					
PBAT	0.629	0.154	0.617	0.202	–
MCC	–	–	−0.032	–	0.867
PLA × PBAT	−0.168	−0.543	–	−0.514	–
PLA × MCC	−0.071	0.027	–	0.539	0.060
PLA × HA	–	−0.289	–	−0.012	–
PBAT × MCC	−0.315	−0.636	−0.319	−0.871	–
MCC × HA	–	−0.095	–	–	0.007

Models were trained using the DoE dataset and evaluated using an independent validation dataset. Hyperparameters (*α*, l_1_ ratio) were optimized using a tolerance of 10^−5^ and a maximum iteration limit of 10^8^. In the table, “Active coefficients” indicate the quantity of nonzero coefficients after model training, which started with 10 terms—PLA, PBAT, MCC, HA, PLA × PBAT, PLA × MCC, PLA × HA, PBAT × MCC, PBAT × HA, and MCC × HA—and was reduced during regularization. *nRMSE* denotes normalized *RMSE*, which was calculated by normalizing the *RMSE* using the experimental range of the response variable and then multiplying by 100%. Standardized regression coefficients are dimensionless. Terms with zero coefficients for all responses (PLA, HA, and PBAT × HA) were omitted. Dashes indicate coefficients equal to zero.

## Data Availability

The original contributions presented in this study are included in the article and Supplementary Materials. Further inquiries can be directed to the corresponding author.

## Supplementary Materials

The following supporting information can be downloaded at: https://www.mdpi.com/article/10.3390/polym18212617/s1. Figure S1. Full mechanical stress-strain curves for all compositions and control samples. Blue represents pristine, green represents UV-aged, and orange represents equilibrated with H_2_O. Each curve was truncated after specimen failure. Figure S2. Full XRD traces for all compositions and control samples with key peaks labeled. The solid black lines represent pristine while the red dashed lines represent UV-aged. Pure MCC and HA powders were only characterized in the pristine condition. Figure S3. SEM images of various magnifications for pristine 8.75/86.25/2.5/2.5 wt% PLA/PBAT/MCC/HA, taken as a representative sample for composite films. Red arrows indicate places where sample damage occurred during scanning. Although lower voltages were attempted, a voltage which did not incur damage could not be performed and prevented us from doing additional characterization such as energy-dispersive X-ray spectroscopy. Figure S4. Full TGA (black) and DTG (red) traces for each composition. Figure S5. DSC thermogram (Endo Up) of commercial MCC powder, indicating that MCC powder does not melt but instead degrades prior to melting. Figure S6. Representative SEM images of various magnifications used for image processing for commercial MCC powder. Figure S7. Representative SEM images of various magnifications used for image processing for commercial HA powder. Figure S8. HA and MCC PSDs used in our compositions. The median and modes are indicated with orange (for HA) and blue (for MCC) dashed lines on the plot. Figure S9. Representative normalized (via min-max normalization) FTIR traces from each sample before (black solid lines) and after UV-accelerated aging (red dashed lines) for each composition. Figure S10. Carbonyl index (CI) and carboxylic acid index (CAI) of biodegradable mulch film formulations before and after UV aging. (a) Carbonyl index (CI) and (b) carboxylic acid index (CAI) calculated from ATR-FTIR spectra using the integrated C=O and COO-H regions normalized to the CH_2_ reference band. Bars represent the mean ± standard deviation of replicate measurements. Asterisks indicate statistically significant differences between pristine and UV-aged samples determined using a paired t-test (* *p* < 0.05, ** *p* < 0.01, *** *p* < 0.001). Figure S11. Diagram of constituents in biodegradable composite. Figure S12. Irradiation profiles for each batch of UV aging experiments. The dotted lines represent the average value used for calculating the exposure time. Figure S13. Accelerated UV aging experiment cycles. Time periods are colored according to the lamp power-yellow regions indicate ON while blue indicates OFF. Temperature profiles and cycles for each batch of UV aging experiments. Note that in Batch 1 the temperature sensor was moved during sample rotation and there was a device power issue that changed the temperature profile briefly for the first day. Table S1. DoE boundary conditions. Table S2. Summary of Characterization Results for Each Run. Table S3. Results After Testing Whether Each Run is Inside or Outside the Convex Hull of the Training Dataset.

## Abbreviations

The following abbreviations are used in this manuscript:

BA	Butylene adipate
BGU	Ben-Gurion University
BT	Butylene terephthalate
D	Crystallite size
DoE	Design of experiments
DSC	Differential scanning calorimetry
DTG	Derivative thermogravimetry
HA	Hydroxyapatite
MCC	Microcrystalline cellulose
M_n_	Number average molecular weight
M_w_	Weight average molecular weight
NJIT	New Jersey Institute of Technology
NSF	National Science Foundation
PBAT	Poly(butylene adipate-*co*-terephthalate)
PLA	Poly(lactic acid)
SEM	Scanning electron microscopy
T_50_	Temperature at which 50% of mass remains (i.e., 50% mass loss)
T_85_	Temperature at which 85% of mass remains (i.e., 15% mass loss), taken to be the onset of decomposition
T_90_	Temperature at which 90% of mass remains (i.e., 10% mass loss)
T_Max_	Temperature of maximum decomposition
*T_g_*	Glass-transition temperature
TGA	Thermogravimetric analysis
ΔH_cc_	Enthalpy of cold crystallization
ΔH^0^_fusion_	Standard heat of fusion for infinitely thick crystalline form
ΔH_m_	Enthalpy of fusion
ΔH_r_	Enthalpic relaxation
ε_Break_	Elongation at break
σ_Break_	Stress at break
*χ*	Percent crystallinity
XRD	X-ray diffraction
