# Supplementary material for: Data-Driven Design of PLA/PBAT/MCC/HA Composites for Biodegradable Mulch Films
Source: Polymers (Basel). 2026 Oct 22;18(21):2617. doi: 10.3390/polym18212617 (PMC13765873; doi:10.3390/polym18212617)
Supplement: Supplementary file 1 [file polymers-18-02617-s001.zip › polymers-4622442-supplementary.pdf]

Supporting Information for Data-Driven Design of PLA/PBAT/MCC/HA Composites for Biodegradable Mulch Films

Nikki Rodriguez [1], Wei Ding [1], Lekhana Sandra [2], Camila Martinez [1], Riddhi Deepak [1], Ilya Gelfand [3], Osnat Gillor [4], Murat Guvendiren [1,5] and Lisa Axe [1,*]

[1] Otto H. York Chemical and Materials Engineering Department, New Jersey Institute of Technology, 138 Warren Street, Newark, NJ 07102, USA; ncr22@njit.edu (N.R.); cm659@njit.edu (C.M.); muratg@njit.edu (M.G.)

[2] Department of Data Science, New Jersey Institute of Technology, 138 Warren Street, Newark, NJ 07102, USA

[3] The French Associates Institute for Agriculture and Biotechnology of Drylands, Ben-Gurion University of the Negev, Midreshet Ben-Gurion 8499000, Israel; igelfand@bgu.ac.il

[4] Zuckerberg Institute for Water Research, Jacob Blaustein Institutes for Desert Research, Ben-Gurion University of the Negev, Sde Boker Campus, Midreshet Ben-Gurion 8499000, Israel; gilloro@bgu.ac.il

[5] Department of Biomedical Engineering, New Jersey Institute of Technology, 138 Warren Street, Newark, NJ 07102, USA

[*] Correspondence: axe@njit.edu

A. Supporting Characterization

Design of Experiment (DoE) parameters are listed in Table S1 for poly(lactic acid) (PLA), poly(butylene adipate-*co*-terephthalate) (PBAT), microcrystalline cellulose (MCC), and hydroxyapatite (HA).

Table S1. DoE boundary conditions.

Factor	Lower Limit	Upper Limit
PLA (wt%)	5	30
PBAT (wt%)	70	95
MCC (wt%)	0	10
HA (wt%)	0	10

Mechanical properties, elongation at break (ε_{Break}) and stress at break (σ_{Break}), were calculated from tensile engineering stress-strain curves. Percent ε_{Break} was calculated using L_{Break}, the extension or gage length of the sample at break (mm), and L_0, the initial gage length (not to be confused with initial grip separation) of the sample at the start of the experiment (mm) (ASTM International 2018) (Equation S1).

$$\varepsilon_{Break} = \frac{L_{Break} - L_0}{L_0} \cdot 100\% \qquad \text{(S1)}$$

σ_{Break} was calculated from F_{Break}, the force when the sample has broken, and A, the cross-sectional area of the sample's gage portion (Equation S2).

$$\sigma_{Break} = \frac{F_{Break}}{A} \qquad \text{(S2)}$$

Full stress-strain curves from which mechanical properties were calculated for all compositions and control samples are shown in Figure S1.

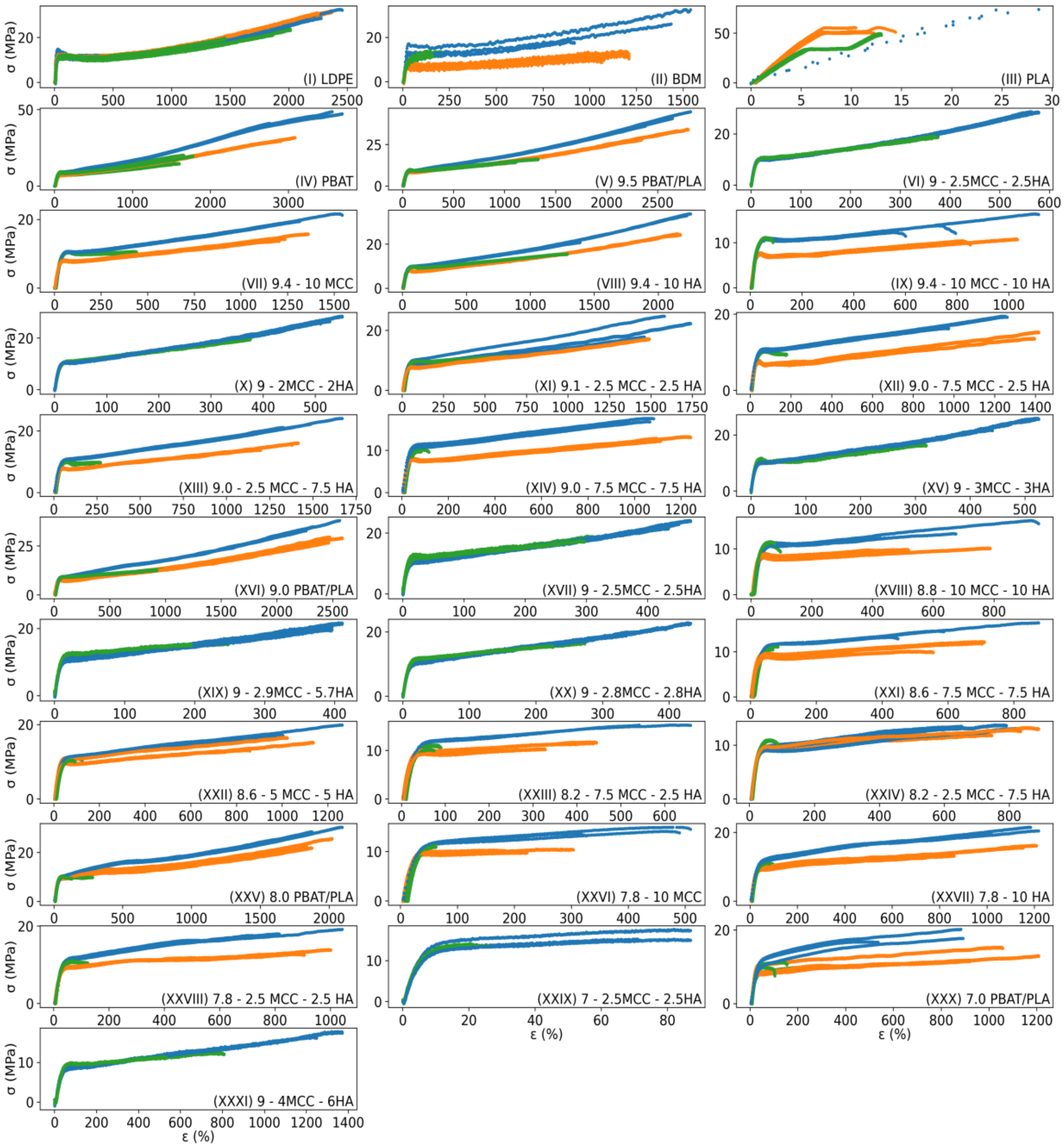

Figure S1. Full mechanical stress-strain curves for all compositions and control samples. Blue represents pristine, green represents UV-aged, and orange represents equilibrated with H_2O. Each curve was truncated after specimen failure.

X-ray diffraction (XRD) diffractograms for the full experimental set are found in Figure S2. Fourier-Transform infrared spectroscopy (FTIR) traces for the full experimental set are found in Figure S9. Carbonyl index (CI) and Carboxylic Acid Index (CAI) results are presented in Figure S10. Scanning electron microscope (SEM) images for the composite film and constituent particles (MCC and HA) can be found in Figures S3, S6, and S7,

respectively. Additionally, the particle size distributions obtained from Figures S6 and S7 can be found in Figure S8. Note that the sample damage incurred during imaging can be seen in Figure S3(g). SEM images (Figures S3, S6, S7) were used to represent the composite (Figure S11). DoE composites are comprised of PLA and PBAT polymer matrix with MCC and HA particles embedded (Figure S11). Figure S5 shows the results of DSC characterization, illustrating that MCC does not melt and instead undergoes significant thermal degradation during the first heating run. DSC traces for MCC were obtained using a DSC 6000 (PerkinElmer Inc, Springfield, IL, USA) and 10-12 mg of sample in a crimp-sealed 50 μL vented aluminum pan, a heating rate of 10 °C·min^{-1}, and under 20 mL·min^{-1} nitrogen flow for all temperature ramps. The temperature program followed: isotherm for 1.0 min at 30°C; heating from 30 to 200°C; isotherm for 3.0 min at 200°C; cooling from 200 to 0°C; and heating from 0 to 200°C. These results were also reproduced at 5 °C·min^{-1}. Full thermogravimetric analysis (TGA) and derivative thermogravimetry (DTG) traces are available in Figure S4. Calculations used in UV aging and associated figures can be found in Section B. The script used to perform Elastic Net Regression can be found in Section C.

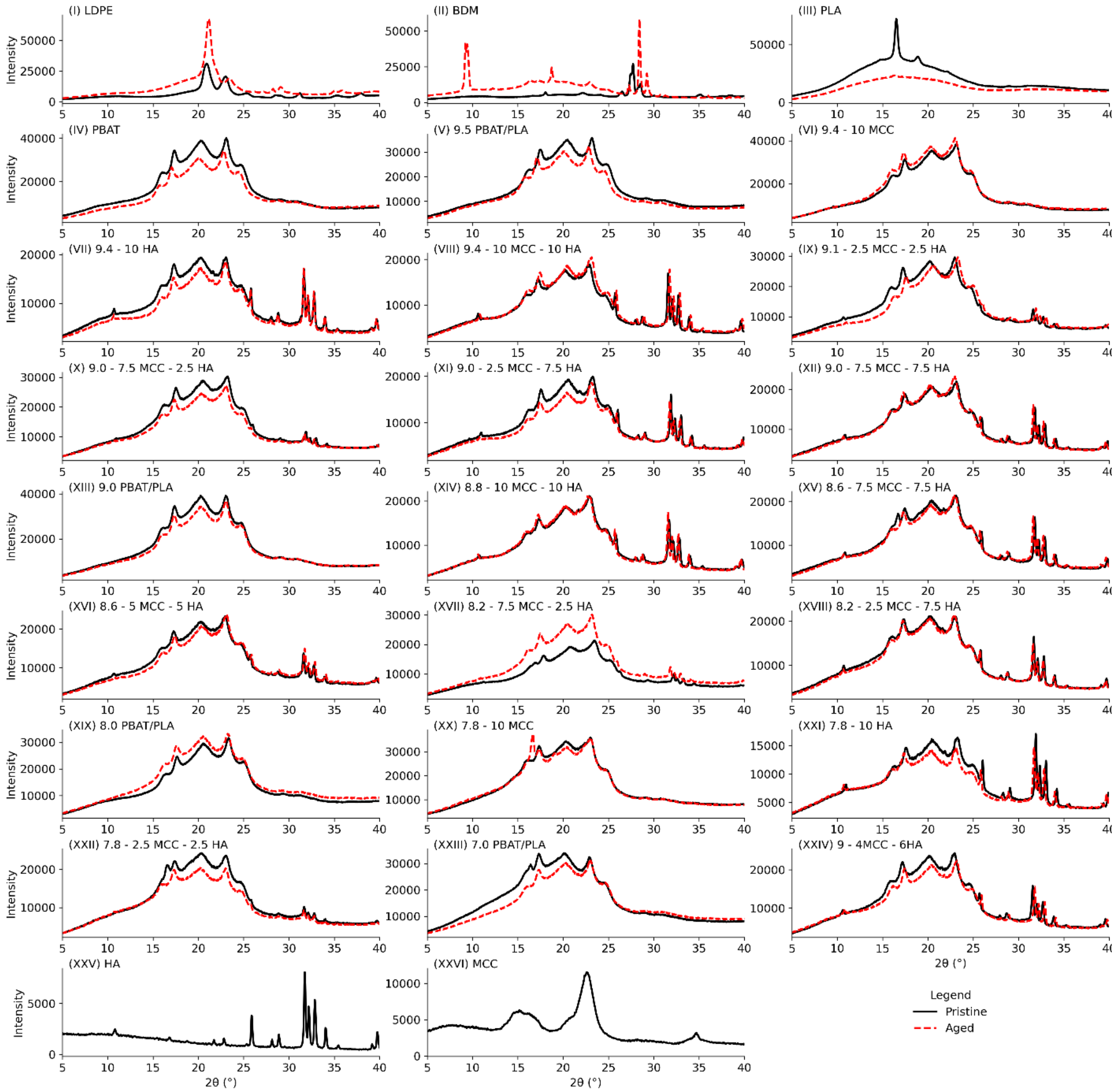

Figure S2 Full XRD traces for all compositions and control samples with key peaks labeled. The solid black lines represent pristine while the red dashed lines represent UV-aged. Pure MCC and HA powders were only characterized in the pristine condition.

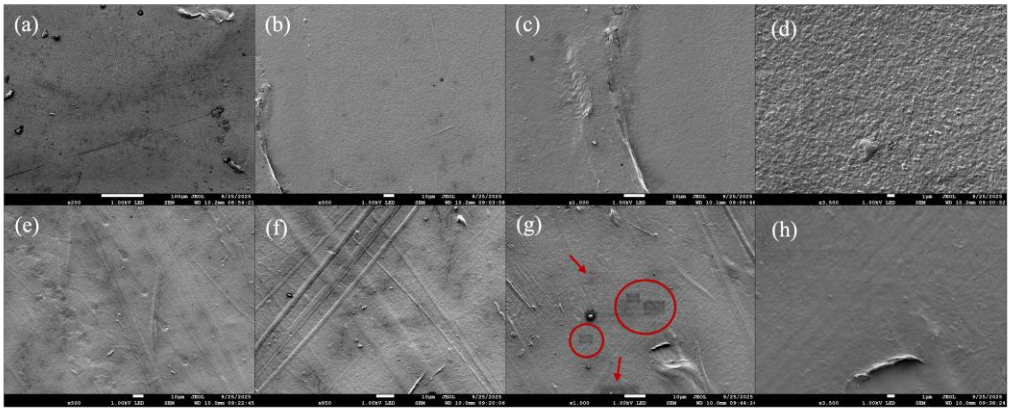

Figure S3 SEM images of various magnifications for pristine 8.75/86.25/2.5/2.5 wt% PLA/PBAT/MCC/HA, taken as a representative sample for composite films. Red arrows indicate places where sample damage occurred during scanning. Although lower voltages were attempted, a voltage which did not incur damage could not be performed and prevented us from doing additional characterization such as energy-dispersive X-ray spectroscopy

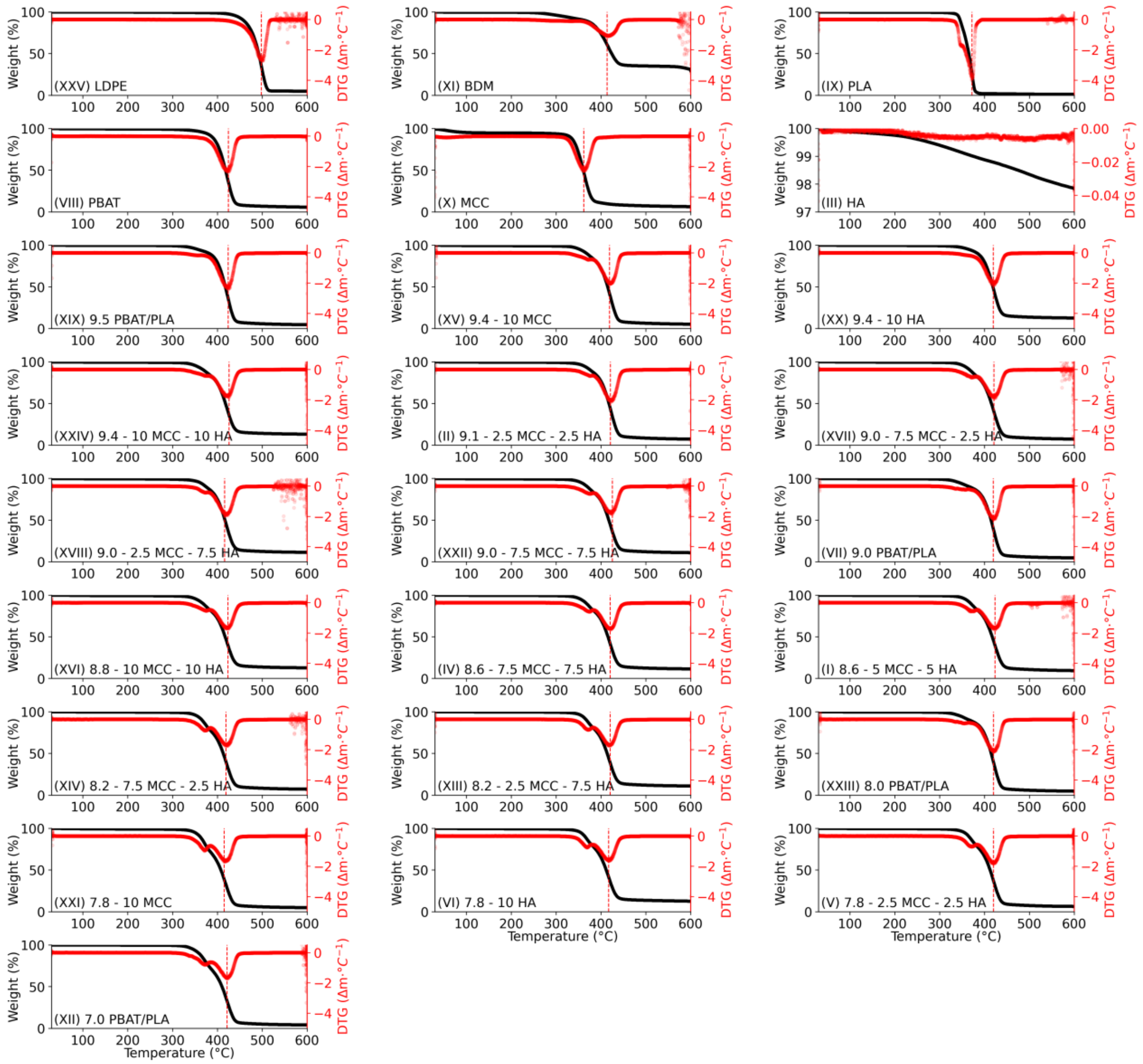

Figure S4 Full TGA (black) and DTG (red) traces for each composition.

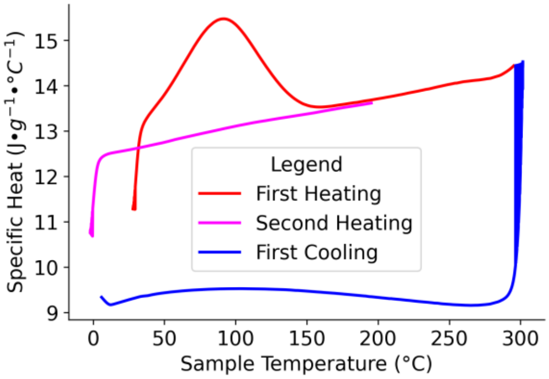

Figure S5 DSC thermogram (Endo Up) of commercial MCC powder, indicating that MCC powder does not melt but instead degrades prior to melting.

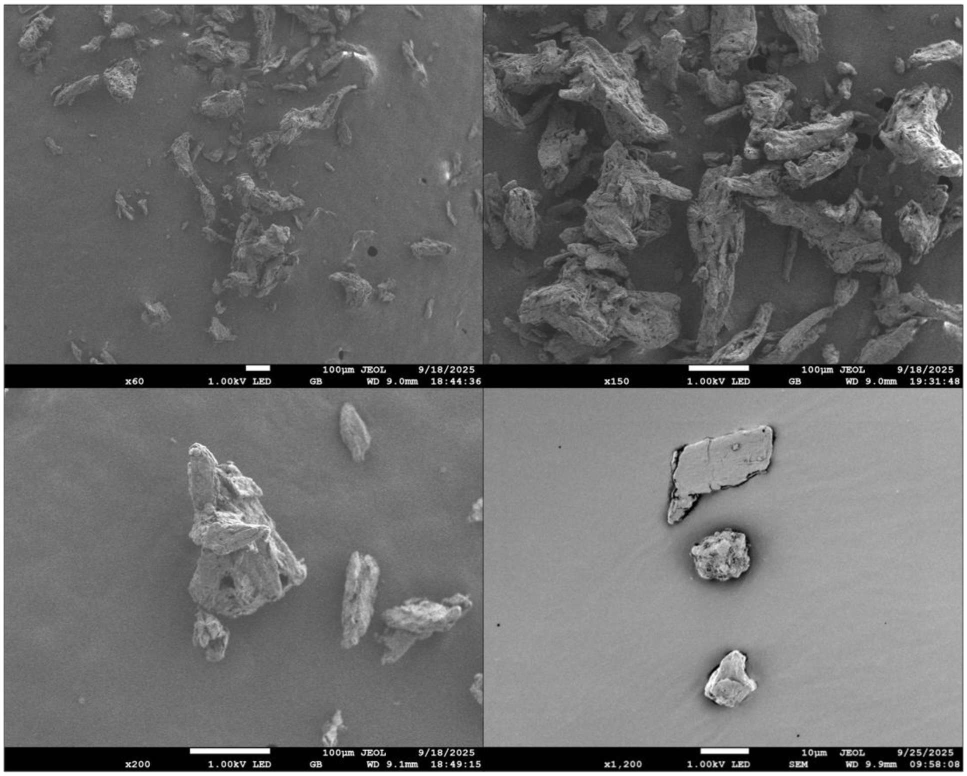

Figure S6 Representative SEM images of various magnifications used for image processing for commercial MCC powder.

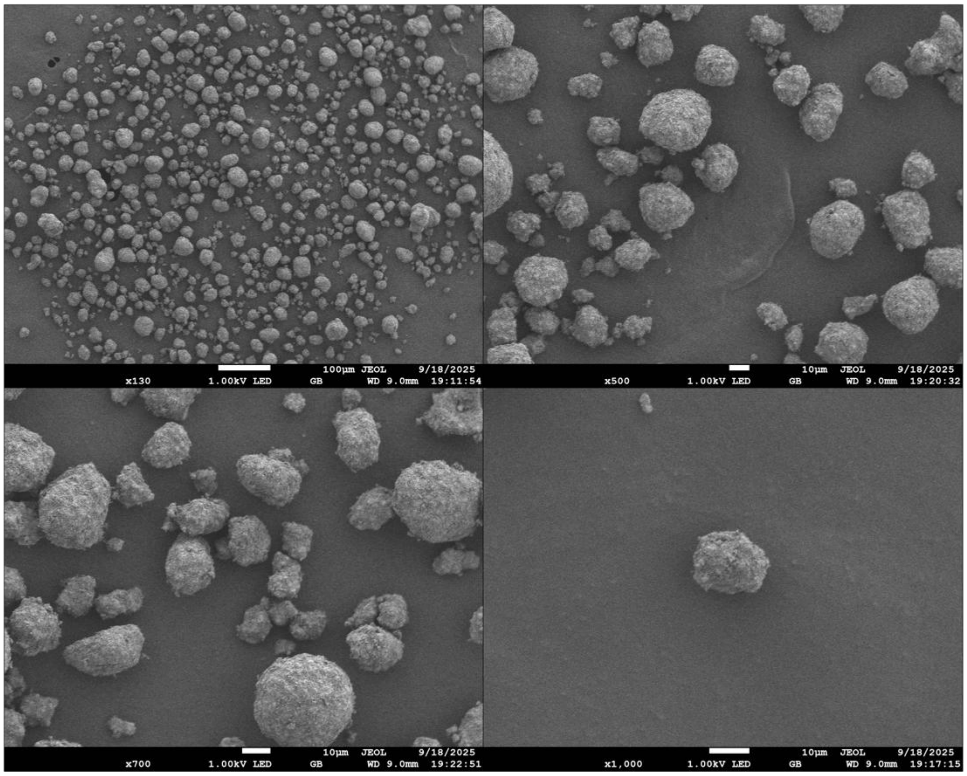

Figure S7 Representative SEM images of various magnifications used for image processing for commercial HA powder.

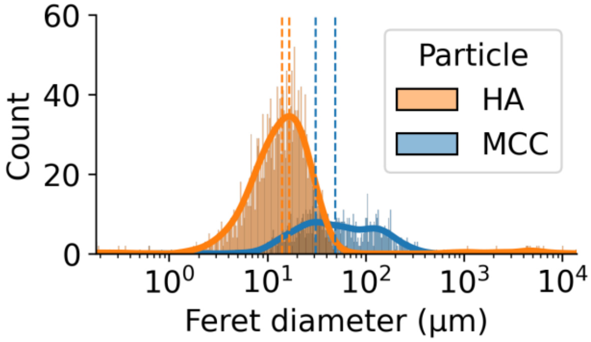

Figure S8 HA and MCC PSDs used in our compositions. The median and modes are indicated with orange (for HA) and blue (for MCC) dashed lines on the plot.

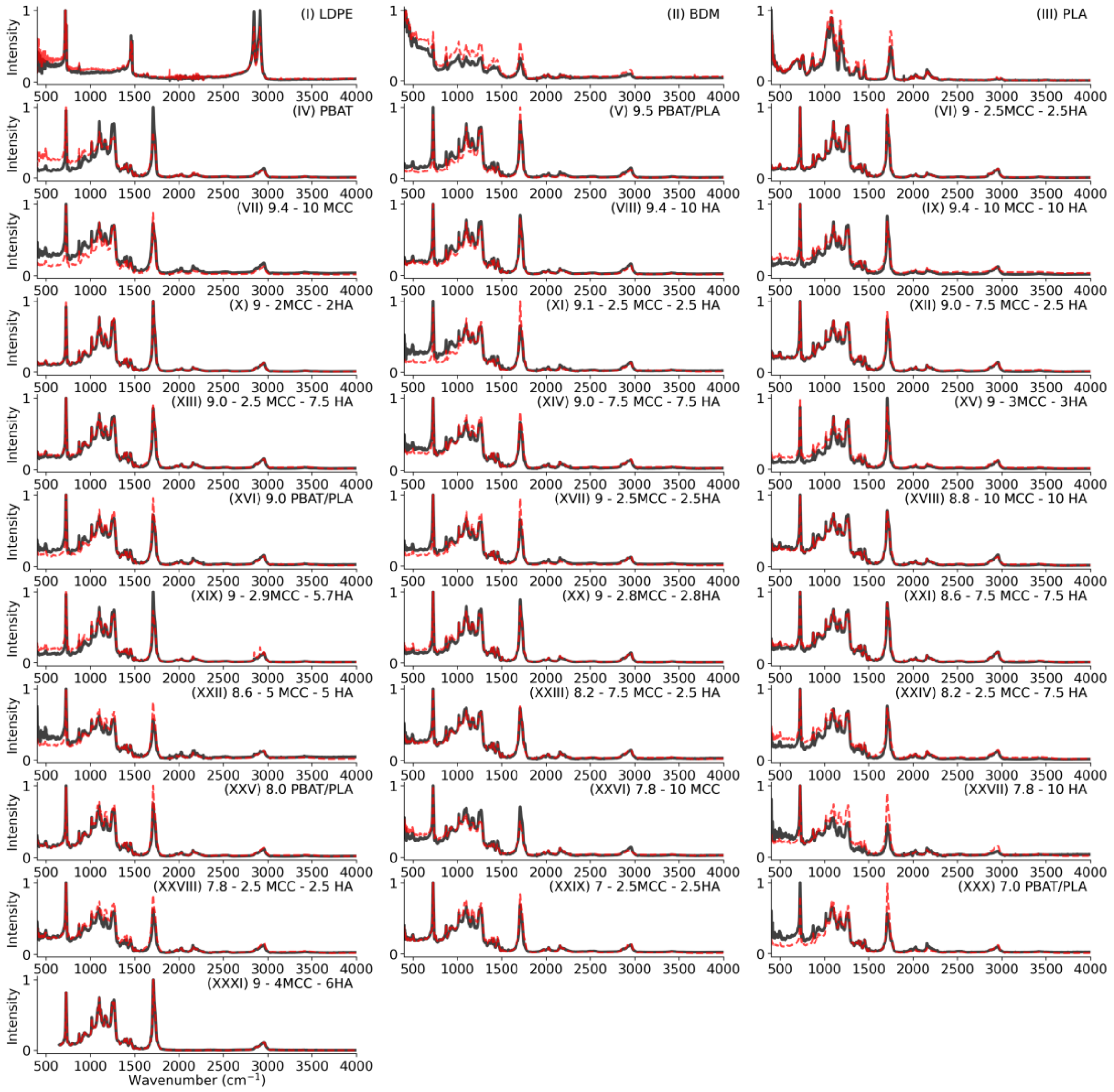

Figure S9 Representative normalized (via min-max normalization) FTIR traces from each sample before (black solid lines) and after UV-accelerated aging (red dashed lines) for each composition.

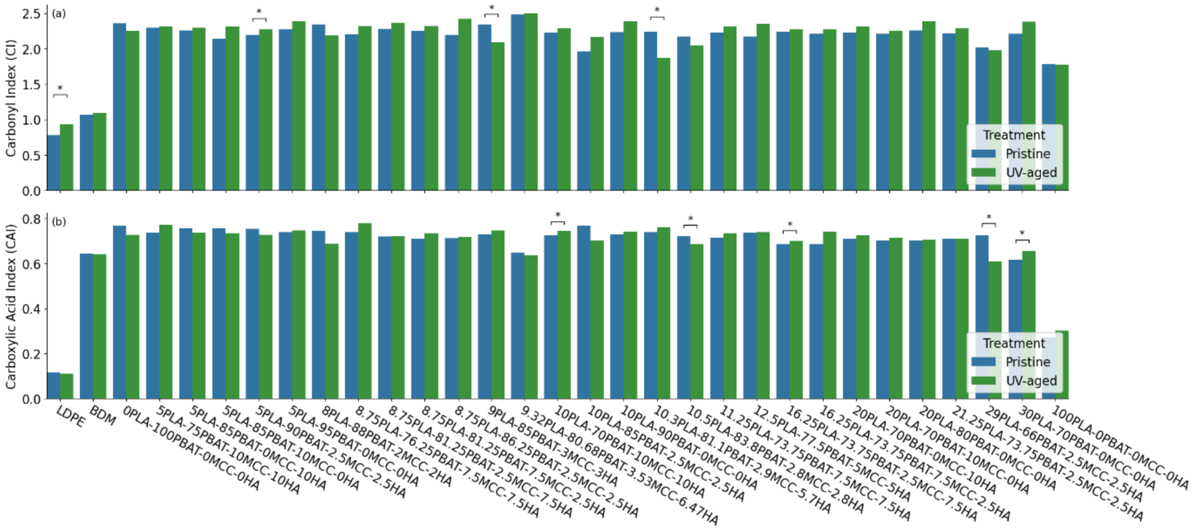

Figure S10 Carbonyl index (CI) and carboxylic acid index (CAI) of biodegradable mulch film formulations before and after UV aging. (a) Carbonyl index (CI) and (b) carboxylic acid index (CAI) calculated from ATR-FTIR spectra using the integrated C=O and COO-H regions normalized to the CH_2 reference band. Bars represent the mean ± standard deviation of replicate measurements. Asterisks indicate statistically significant differences between pristine and UV-aged samples determined using a paired t-test (* $p < 0.05$, ** $p < 0.01$, *** $p < 0.001$).

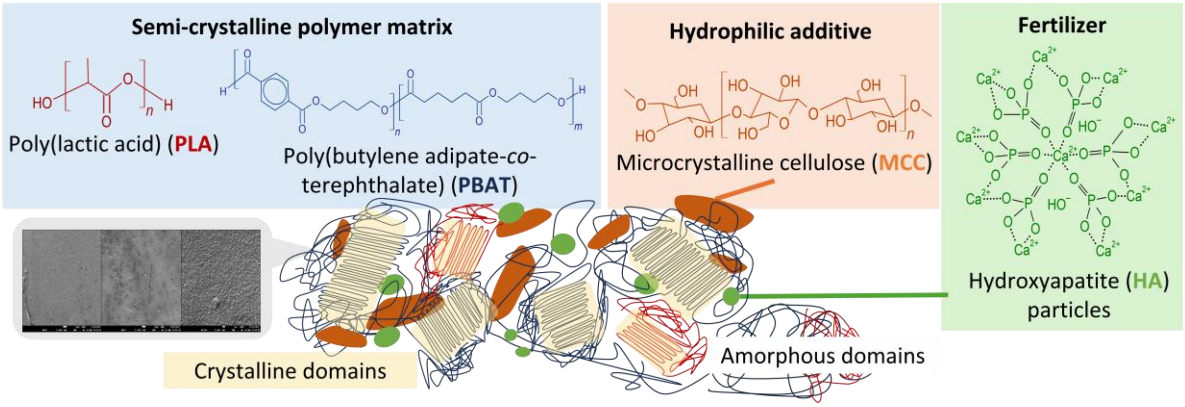

Figure S11 Diagram of constituents in biodegradable composite.

Table S2. Summary of Characterization Results for Each Run.

Run ID	PLA/PBAT/MCC/HA	ε_{Break}	σ_{Break}	Water Abs.	Δ(CI)	Δ(CAI)	χ	D	Char	Moisture	T_{90}	T_{85}	T_{50}	T_{max}
	wt%	%	MPa	%	%	%	%	nm	%	%	°C	°C	°C	°C
1[D]	30/70/0/ 0	770.1 ± 202.6	18.1 ± 1.8	0.4 ± 0.02	9.64 ± 20.44	6.38 ± 2.32	3.5	95.3	4.1	0.050	358.8	367.0	410.1	421.6
2[D]	21.25/73.75/2.5/2.5	831.5 ± 203.7	17.8 ± 1.3	0.66 ± 0.27	3.44 ± 5.19	0.08 ± 3.24	5.1	113.7	6.4	0.160	365.7	373.0	412.5	420.5
3[D]	20/70/10/0	473.3 ± 39.6	13.6 ± 1.1	1.74 ± 0.03	2.53 ± 1.67	6.97 ± 10.3	4.6	53.4	4.9	0.440	361.4	368.0	407.7	415.3
4[D]	20/70/0/10	1240.4 ± 311.1	19.4 ± 1.8	0.5 ± 0.07	2.26 ± 0.95	-6.36 ± 5.74	8.1	151.4	12.5	0.200	364.7	371.6	411.7	417.4
5[D]	16.25/73.75/7.5 / 2.5	613.5 ± 131.36	16.7 ± 2.81	1.13 ± 0.04	13.52 ± 1.96	-2.73 ± 2.7	4.9	136.0	7.1	0.240	365.6	372.9	412.9	419.2
6[D]	16.25/73.75/2.5/7.5	757.8 ± 70.1	18.1 ± 2.7	1.39 ± 0.02	0.19 ± 0.69	4.34 ± 2.36	9.0	166.1	10.9	0.240	367.6	375.1	413.8	420.8
7[D]	12.5/77.5/5/5	1184.8 ± 263.43	21.5 ± 2.3	1.06 ± 0.01	4.53 ± 3.4	4.37 ± 6.25	5.6	86.2	9.4	0.170	368.7	378.4	417.2	423.9
8[D]	11.25/73.75/7.5/7.5	553.8 ± 109.8	16.1 ± 3.5	1.73 ± 0.04	4.08 ± 1.44	3.3 ± 3.74	7.5	158.7	11.4	0.240	366.6	376.4	415.5	420.5
9[D]	10/70/10/10	482.7 ± 38.5	15.8 ± 1.2	2.2 ± 0.02	1.64 ± 1.64	-1.0 ± 2.42	7.8	71.9	12.7	0.420	367.6	377.5	417.1	423.5
10[D]	8.75/81.25/7.5/2.5	1164.1 ± 165.0	18.2 ± 1.8	1.42 ± 0.14	3.7 ± 5.72	-3.86 ± 3.84	5.6	105.5	7.3	0.200	367.5	377.8	415.5	421.1
11[D]	8.75/86.25/2.5/2.5	1593.8 ± 139.5	21.5 ± 3.6	1.1 ± 0.59	11.6 ± 15.36	0.7 ± 0.58	5.8	139.8	7.3	0.190	369.4	388.3	417.8	421.1
12[D]	8.75/81.25/2.5/7.5	1123.3 ± 243.1	18.0 ± 2.2	1.4 ± 0.04	5.1 ± 9.75	-6.6 ± 7.77	6.9	146.5	11.3	0.250	368.4	386.4	416.7	416.3
13[D]	8.75/76.25/7.5/7.5	530.5 ± 156.3	14.3 ± 1.9	2.1 ± 0.08	-0.4 ± 3.23	-6.8 ± 6.77	5.1	145.7	11.0	0.310	369.7	379.9	417.4	425.8

14[D]	5/75/10/10	837.1 ± 260.0	13.3 ± 2.6	1.8 ± 0.13	0.87 ± 0.84	-2.68 ± 3.58	4.8	129.1	13.2	0.340	368.3	381.5	417.8	426.1
15[D]	5/95/0/0	2616.0 ± 222.33	40.5 ± 4.3	0.3 ± 0.04	4.53 ± 9.91	1.34 ± 1.37	6.0	30.5	4.5	0.170	368.6	393.1	417.6	423.9
16[D]	5/85/0/10	1954.9 ± 490.2	29.1 ± 7.2	0.38 ± 0.14	1.68 ± 1.02	-8.81 ± 6.75	6.9	158.5	12.6	0.230	368.7	394.3	418.9	419.9
17[D]	5/85/10/0	1362.3 ± 163.9	19.8 ± 1.3	1.74 ± 0.02	8.75 ± 6.97	-2.65 ± 5.3	5.8	35.2	5.2	0.360	366.4	379.9	414.9	419.1
LDPE	N/A	2295.7 ± 152.8	29.5 ± 2.3	0.3 ± 0.09	19.6 ± 2.2	-3.5 ± 7.15	27.2	119.5	5.0	0.033	454.7	466.7	492.0	498.2
BDM	N/A	1298.0 ± 332.5	25.4 ± 7.4	-0.9 ± 0.55	2.6 ± 2.9	-0.3 ± 4.4	19.5	209.0	29.0	0.219	356.0	382.4	423.9	413.9
18[C]	100/0/0/0	2023.3 ± 418.7	61.9 ± 3.2	0.3 ± 0.1	-0.92 ± 5.97	-2.74 ± 7.81	4.0	106.6	1.1	0.178	337.3	348.7	365.0	372.0
19[C]	10/90/0/0	2384.0 ± 158.5	35.2 ± 2.6	0.46 ± 0.05	8.20 ± 12.4	1.81 ± 2.62	5.8	35.9	4.7	0.241	366.9	383.9	414.7	420.0
20[C]	0/100/0/0	3338.3 ± 504.5	45.5 ± 4.1	0.4 ± 0.07	-4.7 ± 3.48	-5.2 ± 5.23	6.5	36.2	5.4	0.343	372.5	395.5	418.0	424.4
21[C]	0/0/100/0	–	–	–	–	–	10.5	28.5	6.3	4.67	331.28	339.27	360.58	361.57
22[C]	0/0/0/100	–	–	–	–	–	39.1	175.2	97.8	0.111	Did not decompose			
23[V]	10.5/83.8/2.8/2.8	523.2 ± 71.3	26.0 ± 2.0	0.97 ± 0.04	15.41 ± 1.64	1.99 ± 2.84	–	–	–	–	–	–	–	–
24[V]	10.3/81.1/2.9/5.7	371.1 ± 12.1	23.0 ± 1.5	1.41 ± 0.05	3.41 ± 4.3	-4.77 ± 8.14	–	–	–	–	–	–	–	–
25[V]	5/90/2.5/2.5	492.1 ± 56.8	22.1 ± 1.6	0.97 ± 0.02	3.92 ± 10.54	1.97 ± 10.48	–	–	–	–	–	–	–	–
26[V]	8/88/2/2	569.5 ± 25.1	24.9 ± 0.16	1.01 ± 0.01	3.71 ± 8.15	-1.45 ± 4.35	–	–	–	–	–	–	–	–
27[V]	9/85/3/3	416.2 ± 46.8	25.5 ± 0.23	0.68 ± 0.11	10.05 ± 1.27	0.93 ± 3.84	–	–	–	–	–	–	–	–

28[V]	10/85/2.5/2.5	451.6 ± 18.1	22.7 ± 1.1	0.99 ± 0.01	10.47 ± 10.63	0.07 ± 10.55	–	–	–	–	–	–	–	–
29[V]	29/66/2.5/2.5	81.9 ± 8.68	15.8 ± 1.4	0.98 ± 0.09	-1.44 ± 9.91	-1.44 ± 5.51	–	–	–	–	–	–	–	–
30[V]	9.32/80.68/3.53/6.47	1227.1 ± 127.7	16.2 ± 1.0	0.99 ± 0.02	–	–	–	–	–	–	–	–	–	–

"–" indicates parameters that were not calculated or characterized for that particular composition.

Table S3. Results After Testing Whether Each Run is Inside or Outside the Convex Hull of the Training Dataset.		
Run ID	PLA/PBAT/MCC/HA (wt%)	Convex Hull
1^D	30/70/0/ 0	Hull Points (Training data)
2^D	21.25/73.75/2.5/2.5	
3^D	20/70/10/0	
4^D	20/70/0/10	
5^D	16.25/73.75/7.5 / 2.5	
6^D	16.25/73.75/2.5/7.5	
7^D	12.5/77.5/5/5	
8^D	11.25/73.75/7.5/7.5	
9^D	10/70/10/10	
10^D	8.75/81.25/7.5/2.5	
11^D	8.75/86.25/2.5/2.5	
12^D	8.75/81.25/2.5/7.5	
13^D	8.75/76.25/7.5/7.5	
14^D	5/75/10/10	
15^D	5/95/0/0	
16^D	5/85/0/10	
17^D	5/85/10/0	
LDPE	N/A	N/A
BDM	N/A	N/A
18^C	100/0/0/0	Outside
19^C	10/90/0/0	Inside
20^C	0/100/0/0	Outside
21^C	0/0/100/0	Outside
22^C	0/0/0/100	Outside
23^V	10.5/83.8/2.8/2.8	Outside
24^V	10.3/81.1/2.9/5.7	Inside
25^V	5/90/2.5/2.5	Inside
26^V	8/88/2/2	Inside
27^V	9/85/3/3	Inside
28^V	10/85/2.5/2.5	Inside
29^V	29/66/2.5/2.5	Outside
30^V	9.32/80.68/3.53/6.47	Inside

Points were determined to be within or outside the convex hull of hull points (training data, runs 1^D – 17^D) using the following system of equations:

$$\begin{cases} \boldsymbol{x_{new}} = \lambda_1 x_1 + \lambda_2 x_2 + \cdots \lambda_N x_N \\ \lambda_i \geq 0, i = 1, \ldots, \mathrm{N} \\ \sum_{i=1}^{N} \lambda_i = 1 \end{cases} \tag{S3}$$

where N = 17 for this study, $\mathbf{x}_{\mathbf{new}}$ is the candidate formulation being evaluated (e.g. Run 23[V]), x_i is the composition vector of DoE run i (i.e. the PLA, PBAT, MCC, and HA concentrations), and λ_i is the nonnegative weight assigned to DoE run i. If a feasible set of weights exists, the formulation lies within the DoE convex hull; otherwise, it lies outside the convex hull. Testing whether the runs were within the convex hull of the training dataset was done using system of equations S3 and using Python (Version 3.10.9, Anaconda Inc, Austin, Texas, USA) SciPy library. Results are summarized in Table S3. The script used is as follows:

```
results = []; df=sample_list.copy()
doe_points = df.loc[df["Run Type"].isin(["DOE Vertex", "DOE Center"]),["PLA", "PBAT", "MCC", "HA"]].to_numpy()
runs_to_check = df.loc[df["Run Type"].isin(["Control", "Validation"]),["PLA", "PBAT", "MCC", "HA"]]

def convex_hull_check(point, hull_points, tolerance=1e-7):
    point = np.asarray(point, dtype=float)
    hull_points = np.asarray(hull_points, dtype=float)
    n_runs = len(hull_points)
    A_eq = np.vstack([hull_points.T, np.ones(n_runs)]) # matrix with the coefficients for solving for PLA, PBAT, MCC, HA, and sum of coefficients
    b_eq = np.append(point, 1.0) # vector of target values, the PLA/PBAT/MCC/HA/1 (sum of the coeffficients)
    result = linprog(c=np.zeros(n_runs), A_eq=A_eq, b_eq=b_eq, bounds=[(0, None)] * n_runs, method="highs") # bounds(0, None) makes sure the coefficients are positive
    if not result.success: return False, None, np.nan
    reconstructed_point = result.x @ hull_points
    max_error = np.max(np.abs(reconstructed_point - point))
    inside = (max_error <= tolerance and np.all(result.x >= -tolerance) and abs(result.x.sum() - 1) <= tolerance)
    return inside, result.x, max_error

for _, row in runs_to_check.iterrows():
    point = row[components].to_numpy(dtype=float)
```

```
    inside, weights, max_error = convex_hull_check(point=point,
    hull_points=doe_points)
    results.append({"PLA": row["PLA"], "PBAT": row["PBAT"], "MCC": row["MCC"],
    "HA": row["HA"], "Inside convex hull": inside, "Maximum reconstruction error":
    max_error})

hull_results = pd.DataFrame(results)
print(hull_results)
```

B. Ultraviolet Aging Calculations

UV aging studies were done in "batches" due to space and sample preparation constraints. The target exposure was calculated using:

$$(1000\ kW \cdot hour \cdot m^{-2} \cdot year^{-1}) \cdot (0.05) \cdot \frac{3\ months}{12\ months \cdot year^{-1}} = 12.5\ kW \cdot hour \cdot m^{-2}$$

Irradiation time for each batch of UV aging was as follows:

$$Batch\ 1\text{:}\ \frac{12.5\ kW \cdot hour \cdot m^{-2}}{0.0806394\ kW \cdot m^{-2}} \cdot \frac{1\ day}{16\ hours\ LIGHT\ ON} = 9.688\ days$$

$$Batch\ 2\text{:}\ \frac{12.5\ kW \cdot hour \cdot m^{-2}}{0.095638\ kW \cdot m^{-2}} \cdot \frac{1\ day}{16\ hours\ LIGHT\ ON} = 8.169\ days$$

$$Batch\ 3\text{:}\ \frac{12.5\ kW \cdot hour \cdot m^{-2}}{0.091861\ kW \cdot m^{-2}} \cdot \frac{1\ day}{16\ hours\ LIGHT\ ON} = 8.505\ days$$

$$Batch\ 4\text{:}\ \frac{12.5\ kW \cdot hour \cdot m^{-2}}{0.096394\ kW \cdot m^{-2}} \cdot \frac{1\ day}{16\ hours\ LIGHT\ ON} = 8.105\ days$$

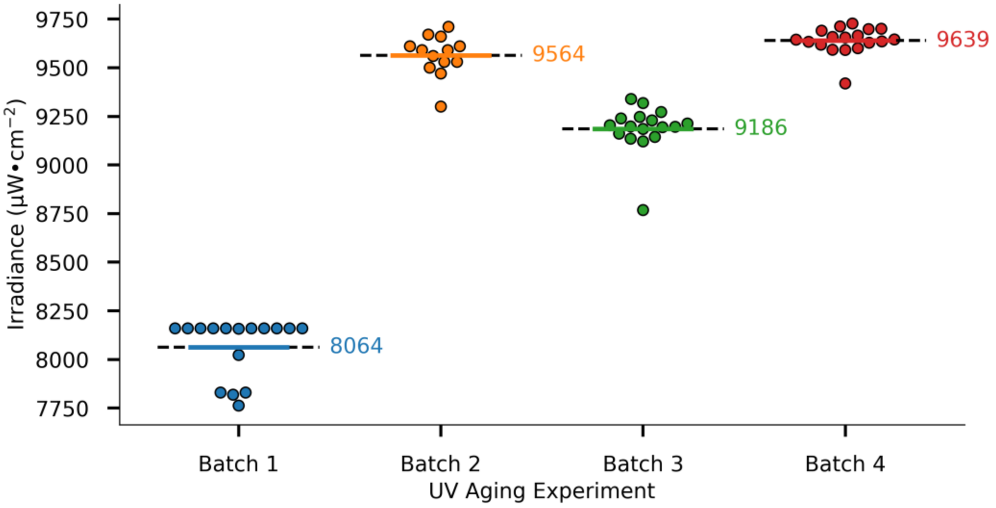

Figure S12 Irradiation profiles for each batch of UV aging experiments. The dotted lines represent the average value used for calculating the exposure time.

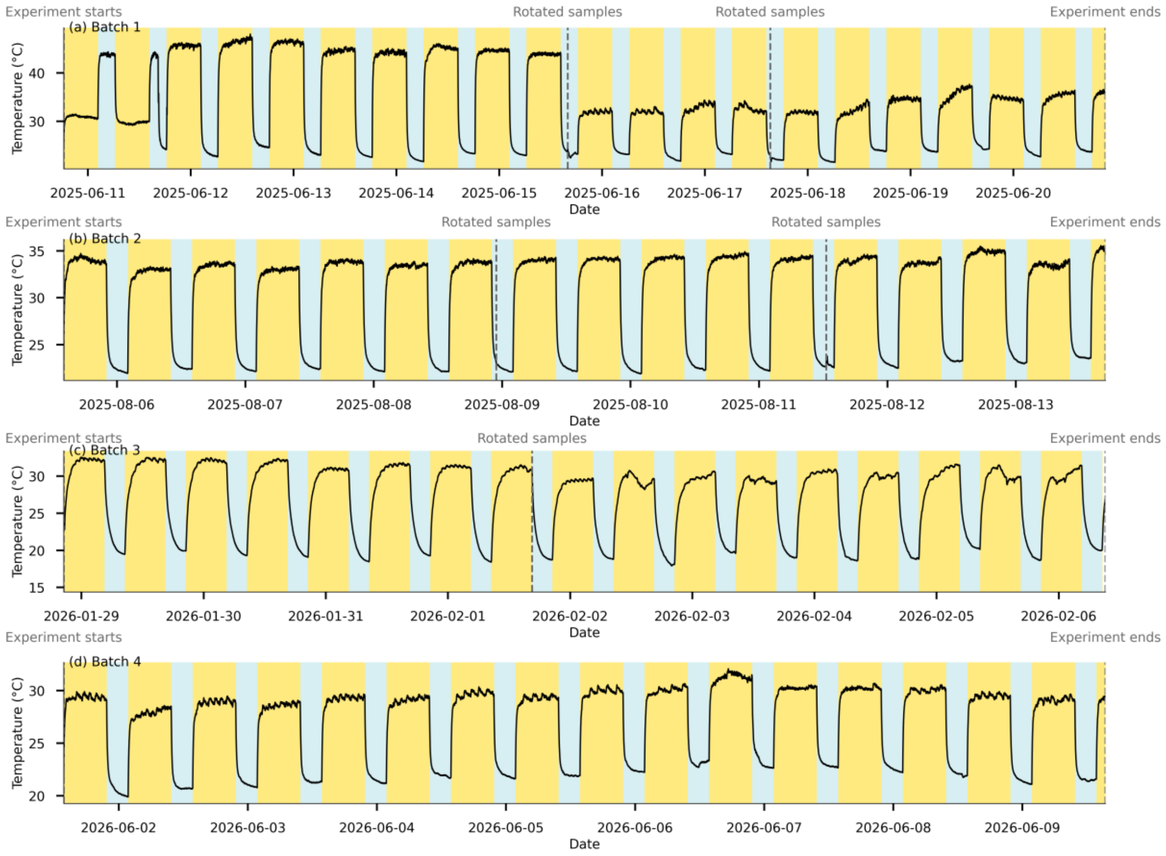

Figure S13 Accelerated UV aging experiment cycles. Time periods are colored according to the lamp power - yellow regions indicate ON while blue indicates OFF. Temperature profiles and cycles for each batch of UV aging experiments. Note that in Batch 1 the temperature sensor was moved during sample rotation and there was a device power issue that changed the temperature profile briefly for the first day.

B. Elastic Net Regression

```
import platform
import numpy as np,  pandas as pd,  seaborn as sns, matplotlib.pyplot as plt
from sklearn.pipeline import Pipeline
from sklearn.preprocessing import PolynomialFeatures, StandardScaler
from sklearn.linear_model import ElasticNetCV
from sklearn.compose import TransformedTargetRegressor
from sklearn.metrics import root_mean_squared_error, r2_score
from scipy.optimize import minimize

##############################################################################################
#################################
# Setting font size
plt.rc('font', size=18) # Controls default text sizes
plt.rc('axes', titlesize=18, labelsize=18) # Sets axes title and label sizes
plt.rc('xtick', labelsize=18)

##############################################################################################
#################################
# Paths
if platform.system() == "Darwin":
   rootdir = r"/Users/nikki/Library/CloudStorage/OneDrive-NJIT/NJIT/Research - Dr. Axe/Projects/Design
of Experiment/Data"
   delimiter = "/"
elif platform.system() == "Windows":
   rootdir = r"C:\Users\nikki\OneDrive - NJIT\NJIT\Research - Dr. Axe\Projects\Design of
Experiment\Data"
   delimiter = "\\"
else:
   rootdir = "."
   delimiter = "/"

df = pd.read_csv(rootdir + delimiter + 'data_compiled.csv')
df_train = df[df['Run Type'].isin(['DOE Vertex', 'DOE Center'])].copy()
df_test = df[df['Run Type'].isin(['Validation', 'Optimized'])].copy()

##############################################################################################
#################################
# Goals Format: response_name: (goal_type, lower_limit, target_or_upper, weight)
```

```
goals = {"Pristine_EB": ("max", 350, 420, 1), "Aged_EB": ("max", 350, 420, 1), "Pristine_SB": ("max", 9, 18, 1),
"Aged_SB": ("max", 9, 18, 1), "WaterAbs": ("max", 0.8, 1.0, 1)}

##############################################################################################
##################################
# Bounds + constraints + constants etc etc etc
#PLA, PBAT, MCC, HA
bounds = [(0, 30), (70, 90), (0, 10),(0, 10)]
x0 = [8.75, 86.25, 2.5, 2.5]
ha_weight = 1
pla_weight = 1  # Weight for maximizing PLA in desirability (0 = ignore, higher = more PLA)
tol = 1e-5
max_iter = int(1e8)
line_kws = {'linestyle': '--', 'linewidth': 1}
sum_constraint = {"type": "eq", "fun": lambda x: x[0] + x[1] + x[2] + x[3] - 100}
max_powder_loading = {"type": "ineq", "fun": lambda x: 10 - (x[2] + x[3])}
constraints = [sum_constraint, max_powder_loading]
letters = "abcdefghijklmnopqrstuvwxyz"
dpi=300

##############################################################################################
##################################
# Model class
class ElasticNetDOE:
    def __init__(self, predictors=("PLA", "PBAT", "MCC", "HA"), poly_interaction_only=False):
        self.predictors = list(predictors)
        self.poly_interaction_only = poly_interaction_only
        self.models = {}
        self.best_params_ = {}
        self.metrics_ = {}
        self.predictions_ = {}

    def _build_model(self):
        pipe = Pipeline([
            ("poly", PolynomialFeatures(
                degree=2,
                interaction_only=True,
                include_bias=False)),
            ("scaler", StandardScaler()),
            ("enet", ElasticNetCV(
                l1_ratio=np.linspace(0.5, 1.0, 10),
                alphas=np.logspace(-6, -1, 10),
                fit_intercept=True,
                tol=tol,
                max_iter=max_iter,
                n_jobs=-1))])

        return TransformedTargetRegressor(
```

```
        regressor=pipe,
        transformer=StandardScaler())

def fit(self, df_train, df_test, responses, exclude_controls=False):
    train = df_train.copy().dropna(subset=self.predictors + responses).reset_index(drop=True)
    test = df_test.copy().dropna(subset=self.predictors + responses).reset_index(drop=True)

    X_train = train[self.predictors]
    X_test = test[self.predictors]

    for resp in responses:
        y_train = train[resp]
        y_test = test[resp]

        model = self._build_model()
        model.fit(X_train, y_train)

        y_train_pred = model.predict(X_train)
        y_test_pred = model.predict(X_test)

        enet_step = model.regressor_.named_steps["enet"]

        self.models[resp] = model
        self.best_params_[resp] = {
            "alpha": enet_step.alpha_,
            "l1_ratio": enet_step.l1_ratio_}

        self.metrics_[resp] = {
            "train_R2": r2_score(y_train, y_train_pred),
            "test_R2": r2_score(y_test, y_test_pred),
            "train_RMSE": root_mean_squared_error(y_train, y_train_pred),
            "test_RMSE": root_mean_squared_error(y_test, y_test_pred)}

        self.predictions_[resp] = {
            "y_train": y_train.values,
            "y_train_pred": y_train_pred,
            "y_test": y_test.values,
            "y_test_pred": y_test_pred}

    return self

def predict_responses(self, x):
    x_df = pd.DataFrame([x], columns=self.predictors)
    preds = {}
    for resp, model in self.models.items():
        preds[resp] = float(model.predict(x_df)[0])
    return preds
```

```
    def predict_dataframe(self, new_df):
        out = new_df.copy()
        X_new = out[self.predictors]
        for resp, model in self.models.items():
            out[resp] = model.predict(X_new)
        return out

    def _individual_desirability(self, y, goal_type, low, high, weight=1):
        if high == low: return 0.0
        if goal_type == "max":
            if y <= low: return 0.0
            elif y >= high: return 1.0
            else: return ((y - low) / (high - low)) ** weight
        elif goal_type == "min":
            if y >= high: return 0.0
            elif y <= low: return 1.0
            else: return ((high - y) / (high - low)) ** weight
        else: raise ValueError(f"Unsupported goal type: {goal_type}")

    def desirability(self, x, goals, ha_weight=0.0, pla_weight=0.0):
        preds = self.predict_responses(x)

        d_list = []
        for resp, (goal_type, low, high, weight) in goals.items():
            y = preds[resp]
            d = self._individual_desirability(y, goal_type, low, high, weight)
            d_list.append(max(d, 1e-12))

        D = np.prod(d_list) ** (1 / len(d_list))

        if ha_weight > 0:
            ha_fraction = x[3] / 10.0
            D *= max(ha_fraction, 1e-12) ** ha_weight

        if pla_weight > 0:
            pla_fraction = x[0] / 30.0  # normalize by max PLA (30 wt%)
            D *= max(pla_fraction, 1e-12) ** pla_weight

        return float(D)

    def optimize_multistart(self, goals, bounds, constraints, x0, ha_weight=0.0, pla_weight=0.0,
n_starts=150, seed=42):
        rng = np.random.default_rng(seed)
        starts = [list(x0)]

        attempts = 0
        while len(starts) < n_starts + 1 and attempts < 50000:
            attempts += 1
```

```
        PLA = rng.uniform(bounds[0][0], bounds[0][1])
        MCC = rng.uniform(bounds[2][0], bounds[2][1])
        HA = rng.uniform(bounds[3][0], bounds[3][1])

        PBAT = 100.0 - PLA - MCC - HA
        if bounds[1][0] <= PBAT <= bounds[1][1]: starts.append([PLA, PBAT, MCC, HA])

    best_result = None
    best_D = -np.inf

    for start in starts:
        res = minimize(
            fun=lambda x: -self.desirability(x, goals, ha_weight=ha_weight, pla_weight=pla_weight),
            x0=np.array(start, dtype=float),
            bounds=bounds,
            constraints=constraints,
            method="SLSQP",
            options={"maxiter": max_iter, "ftol": tol})

        if res.success:
            D = -res.fun
            if D > best_D:
                best_D = D
                best_result = res

    return best_result

def plot_train_test(self, responses):
    n = len(responses)
    fig,axes=plt.subplots(1, n, figsize=(5*n,5))

    if n == 1: axes = [axes]

    for i, (ax, resp) in enumerate(zip(axes, responses)):
        y_train = self.predictions_[resp]["y_train"]
        y_train_pred = self.predictions_[resp]["y_train_pred"]
        y_test = self.predictions_[resp]["y_test"]
        y_test_pred = self.predictions_[resp]["y_test_pred"]

        train_plot = pd.DataFrame({'Experimental': y_train, 'Calculated': y_train_pred})
        test_plot = pd.DataFrame({'Experimental': y_test, 'Calculated': y_test_pred})

        all_vals = np.concatenate([y_train, y_train_pred, y_test, y_test_pred])
        mn = np.min(all_vals)
        mx = np.max(all_vals)

        # add padding
        padding = 0.05 * (mx - mn)
```

```
        mn -= padding
        mx += padding

        sns.regplot(data=train_plot, x='Experimental', y='Calculated', marker='o', color='blue', label='DoE',
ax=ax, line_kws=line_kws)
        sns.regplot(data=test_plot, x='Experimental', y='Calculated', marker='o', color='orange',
label='Validation', ax=ax, line_kws=line_kws)
        title = next((k for k, v in rename_dict.items() if v == resp), resp)
        ax.set_title(f"({letters[i]}) {title}", loc="left")

        ax.set_xlim(mn, mx)
        ax.set_ylim(mn, mx)
        ax.set_aspect('equal', adjustable='box')
        ax.legend()

    plt.tight_layout()
    plt.savefig('fig_predictions.png', dpi=dpi, bbox_inches='tight')
    plt.show()


##################################################################################################
##################################
# Load data
rename_dict = {
    "Pristine ε$_{Break}$ (%)": "Pristine_EB",
    "Pristine σ$_{Break}$ (MPa)": "Pristine_SB",
    "Water Absorption (%)": "WaterAbs",
    "UV-Aged ε$_{Break}$ (%)": "Aged_EB",
    "UV-Aged σ$_{Break}$ (MPa)": "Aged_SB"}

df_train = df_train.rename(columns=rename_dict)
df_test = df_test.rename(columns=rename_dict)

responses = ["Pristine_EB", "Aged_EB", "Pristine_SB", "Aged_SB", "WaterAbs"]
predictors = ["PLA", "PBAT", "MCC", "HA"]

columns = responses + predictors

df_test = df_test[columns].copy()
df_train = df_train[columns].copy()

model = ElasticNetDOE(predictors=("PLA", "PBAT", "MCC", "HA"), poly_interaction_only=False)
model.fit(df_train=df_train, df_test=df_test, responses=responses, exclude_controls=False)

##################################################################################################
##################################
# Print best hyperparameters
print("\nBest hyperparameters from ElasticNetCV:")
```

```
for resp, params in model.best_params_.items():
    print(f"{resp}: alpha={params['alpha']:.6g}, l1_ratio={params['l1_ratio']:.3f}")

##################################################################################################
###################################
# Print metrics
print("\nMetrics:")
metrics_df = pd.DataFrame(model.metrics_).T
print(metrics_df)

##################################################################################################
###################################
# Build export tables
summary_rows = []
coef_rows = []
pred_rows = []

for resp in responses:
    fitted_model = model.models[resp]
    pipe = fitted_model.regressor_
    poly = pipe.named_steps["poly"]
    scaler_x = pipe.named_steps["scaler"]
    enet = pipe.named_steps["enet"]
    scaler_y = fitted_model.transformer_

    feature_names = poly.get_feature_names_out(model.predictors)

    # Summary / hyperparameters / iterations
    n_iter_val = enet.n_iter_
    if np.ndim(n_iter_val) > 0:
        n_iter_max = np.max(n_iter_val)
        n_iter_mean = np.mean(n_iter_val)
    else:
        n_iter_max = n_iter_val
        n_iter_mean = n_iter_val

    summary_rows.append({
        "Response": resp,
        "alpha": enet.alpha_,
        "l1_ratio": enet.l1_ratio_,
        "tol": enet.tol,
        "max_iter": enet.max_iter,
        "n_iter_max": n_iter_max,
        "n_iter_mean": n_iter_mean,
        "n_features_after_poly": len(feature_names),
        "n_nonzero_coefficients": int(np.sum(np.abs(enet.coef_) > 1e-12)),
        "train_R2": model.metrics_[resp]["train_R2"],
        "test_R2": model.metrics_[resp]["test_R2"],
```

```
        "train_RMSE": model.metrics_[resp]["train_RMSE"],
        "test_RMSE": model.metrics_[resp]["test_RMSE"],})

    # Coefficients
    beta_std = enet.coef_.copy()
    x_mean = scaler_x.mean_
    x_scale = scaler_x.scale_

    y_mean = float(np.ravel(scaler_y.mean_)[0])
    y_scale = float(np.ravel(scaler_y.scale_)[0])

    beta_poly = y_scale * beta_std / x_scale
    intercept_poly = y_mean - np.sum(beta_poly * x_mean)

    coef_rows.append({
        "Response": resp,
        "Term": "Intercept",
        "Coef_Standardized": np.nan,
        "Coef_PolynomialUnits": intercept_poly,
        "Nonzero": True})

    for term, b_std, b_poly in zip(feature_names, beta_std, beta_poly):
        coef_rows.append({
            "Response": resp,
            "Term": term,
            "Coef_Standardized": b_std,
            "Coef_PolynomialUnits": b_poly,
            "Nonzero": abs(b_std) > 1e-12})

    # Train/test predictions
    y_train = model.predictions_[resp]["y_train"]
    y_train_pred = model.predictions_[resp]["y_train_pred"]
    y_test = model.predictions_[resp]["y_test"]
    y_test_pred = model.predictions_[resp]["y_test_pred"]

    for actual, pred in zip(y_train, y_train_pred):
        pred_rows.append({"Response": resp, "Dataset": "Train", "Actual": actual, "Predicted": pred,
"Residual": actual - pred})

    for actual, pred in zip(y_test, y_test_pred):
        pred_rows.append({
            "Response": resp,
            "Dataset": "Test",
            "Actual": actual,
            "Predicted": pred,
            "Residual": actual - pred})

summary_df = pd.DataFrame(summary_rows)
```

```
coef_df = pd.DataFrame(coef_rows)
predictions_export_df = pd.DataFrame(pred_rows)

coef_df["Abs_Standardized"] = coef_df["Coef_Standardized"].abs()
coef_df = pd.concat([
    coef_df[coef_df["Term"] == "Intercept"],
    coef_df[coef_df["Term"] != "Intercept"].sort_values(by=["Response", "Abs_Standardized"],
ascending=[True, False])], ignore_index=True)

print("\nModel summary:")
print(summary_df)

print("\nCoefficients:")
print(coef_df)

print("\nTrain/test predictions:")
print(predictions_export_df)

import sys

##################################################################################################
##################################
# Optimization

try:
    print("Starting optimization...", flush=True)

    best_result = model.optimize_multistart(
        goals=goals,
        bounds=bounds,
        constraints=constraints,
        x0=x0,
        ha_weight=ha_weight,
        pla_weight=pla_weight,
        n_starts=150,
        seed=42)

    print(f"Optimization complete. Result found: {best_result is not None}", flush=True)

    if best_result is not None:
        best_x = best_result.x
        best_preds = model.predict_responses(best_x)
        best_D = model.desirability(best_x, goals, ha_weight=ha_weight, pla_weight=pla_weight)

        print("\nBest formulation found:", flush=True)
        print(f"PLA  = {best_x[0]:.3f}", flush=True)
        print(f"PBAT = {best_x[1]:.3f}", flush=True)
        print(f"MCC  = {best_x[2]:.3f}", flush=True)
```

```
        print(f"HA   = {best_x[3]:.3f}", flush=True)
        print(f"Sum  = {sum(best_x):.3f}", flush=True)

        print("\nPredicted responses at optimum:", flush=True)
        for k, v in best_preds.items():
            print(f"{k}: {v:.3f}", flush=True)

        print(f"\nOverall desirability: {best_D:.4f}", flush=True)
    else:
        print("Optimization failed — no feasible solution found.", flush=True)

    model.plot_train_test(responses)

    predicted_new = model.predict_dataframe(df_test[predictors].drop_duplicates())
    print("\nPredictions for validation formulations:", flush=True)
    print(predicted_new, flush=True)

except Exception as e:
    import traceback
    print(f"ERROR: {e}", flush=True)
    traceback.print_exc()
    sys.stdout.flush()
```
